# HLA Ligand Atlas: a benign reference of HLA-presented peptides to improve T-cell-based cancer immunotherapy

**DOI:** 10.1136/jitc-2020-002071

**Published:** 2021-04-15

**Authors:** Ana Marcu, Leon Bichmann, Leon Kuchenbecker, Daniel Johannes Kowalewski, Lena Katharina Freudenmann, Linus Backert, Lena Mühlenbruch, András Szolek, Maren Lübke, Philipp Wagner, Tobias Engler, Sabine Matovina, Jian Wang, Mathias Hauri-Hohl, Roland Martin, Konstantina Kapolou, Juliane Sarah Walz, Julia Velz, Holger Moch, Luca Regli, Manuela Silginer, Michael Weller, Markus W. Löffler, Florian Erhard, Andreas Schlosser, Oliver Kohlbacher, Stefan Stevanović, Hans-Georg Rammensee, Marian Christoph Neidert

**Affiliations:** 1 Department of Immunology, Interfaculty Institute for Cell Biology, University of Tübingen, Tübingen, Germany; 2 Cluster of Excellence iFIT (EXC 2180) "Image-Guided and Functionally Instructed Tumor Therapies", University of Tübingen, Tübingen, Germany; 3 Applied Bioinformatics, Department of Computer Science, University of Tübingen, Tübingen, Germany; 4 DKFZ Partner Site Tübingen, German Cancer Consortium (DKTK), Tübingen, Germany; 5 Department of Obstetrics and Gynecology, University Hospital of Tübingen, Tübingen, Germany; 6 Neuroimmunology and MS Research, Neurology Clinic, University Hospital Zurich, University of Zurich, Zurich, Switzerland; 7 Pediatric Stem Cell Transplantation, University Children’s Hospital Zurich, Zurich, Switzerland; 8 Clinical Neuroscience Center and Department of Neurosurgery, University Hospital and University of Zurich, Zurich, Switzerland; 9 Clinical Collaboration Unit Translational Immunology, German Cancer Consortium (DKTK), University Hospital of Tübingen, Tübingen, Germany; 10 Dr. Margarete Fischer-Bosch Institute of Clinical Pharmacology (IKP) and Robert Bosch Center for Tumor Diseases (RBCT), Stuttgart, Germany; 11 Department of Pathology and Molecular Pathology, University Hospital and University of Zurich, Zurich, Switzerland; 12 Clinical Neuroscience Center and Department of Neurology, University Hospital and University of Zurich, Zurich, Switzerland; 13 Department of General, Visceral and Transplant Surgery, University Hospital of Tübingen, Tübingen, Germany; 14 Department of Clinical Pharmacology, University of Hospital Tübingen, Tübingen, Germany; 15 Institute for Virology and Immunobiology, Julius-Maximilians-University Würzburg, Würzburg, Bayern, Germany; 16 Rudolf Virchow Center - Center for Integrative and Translational Bioimaging, Julius-Maximilians-University Würzburg, Würzburg, Germany; 17 Institute for Bioinformatics and Medical Informatics, University of Tübingen, Tübingen, Germany; 18 Quantitative Biology Center (QBiC), University of Tübingen, Tübingen, Germany; 19 Biomolecular Interactions, Max Planck Institute for Developmental Biology, Tübingen, Germany; 20 Cluster of Excellence Machine Learning in the Sciences (EXC 2064), University of Tübingen, Tübingen, Germany; 21 Institute for Translational Bioinformatics, University Hospital Tübingen, Tübingen, Germany; 22 Department of Neurosurgery, Cantonal Hospital St.Gallen, St.Gallen, Switzerland; 23 Neuroscience Center Zurich (ZNZ), University of Zurich and ETH Zurich, Zurich, Switzerland

**Keywords:** adaptive immunity, immunotherapy, translational medical research, antigens, tumor-associated, carbohydrate, antigen presentation

## Abstract

**Background:**

The human leucocyte antigen (HLA) complex controls adaptive immunity by presenting defined fractions of the intracellular and extracellular protein content to immune cells. Understanding the benign HLA ligand repertoire is a prerequisite to define safe T-cell-based immunotherapies against cancer. Due to the poor availability of benign tissues, if available, normal tissue adjacent to the tumor has been used as a benign surrogate when defining tumor-associated antigens. However, this comparison has proven to be insufficient and even resulted in lethal outcomes. In order to match the tumor immunopeptidome with an equivalent counterpart, we created the HLA Ligand Atlas, the first extensive collection of paired HLA-I and HLA-II immunopeptidomes from 227 benign human tissue samples. This dataset facilitates a balanced comparison between tumor and benign tissues on HLA ligand level.

**Methods:**

Human tissue samples were obtained from 16 subjects at autopsy, five thymus samples and two ovary samples originating from living donors. HLA ligands were isolated via immunoaffinity purification and analyzed in over 1200 liquid chromatography mass spectrometry runs. Experimentally and computationally reproducible protocols were employed for data acquisition and processing.

**Results:**

The initial release covers 51 HLA-I and 86 HLA-II allotypes presenting 90,428 HLA-I- and 142,625 HLA-II ligands. The HLA allotypes are representative for the world population. We observe that immunopeptidomes differ considerably between tissues and individuals on source protein and HLA-ligand level. Moreover, we discover 1407 HLA-I ligands from non-canonical genomic regions. Such peptides were previously described in tumors, peripheral blood mononuclear cells (PBMCs), healthy lung tissues and cell lines. In a case study in glioblastoma, we show that potential on-target off-tumor adverse events in immunotherapy can be avoided by comparing tumor immunopeptidomes to the provided multi-tissue reference.

**Conclusion:**

Given that T-cell-based immunotherapies, such as CAR-T cells, affinity-enhanced T cell transfer, cancer vaccines and immune checkpoint inhibition, have significant side effects, the HLA Ligand Atlas is the first step toward defining tumor-associated targets with an improved safety profile. The resource provides insights into basic and applied immune-associated questions in the context of cancer immunotherapy, infection, transplantation, allergy and autoimmunity. It is publicly available and can be browsed in an easy-to-use web interface at **https://hla-ligand-atlas.org**.

## Introduction

In the past two decades, sequencing the human genome (*genomics*),^1 2^ transcriptome (*transcriptomics*)^3 4^ and proteome (*proteomics*)^5–7^ have been milestones that enable a multidimensional understanding of biological processes. In the context of the immune system, a subsequent *omics* layer can be defined as the human leucocyte antigen (HLA) ligandome or the immunopeptidome, comprizing the entirety of HLA presented peptides. HLA molecules present peptides on the cell surface for recognition by T cells, which were generated and selected to distinguish self from foreign^8^ peptides. Despite HLA-I ligands originating primarily from intracellular proteins, the correlation with their precursors (mRNA transcripts and proteins) is poor,^9–11^ limiting approaches based on *in silico* HLA-binding predictions in combination with transcriptomics and proteomics data alone.^12^

The importance of investigating HLA ligandomes from human healthy and diseased tissues has been well recognized^13–15^ to improve HLA-binding prediction algorithms,^16–18^ and immunogenicity prediction analysis,^19^ but also, to inform precision medicine.^20–22^ Direct evidence of naturally presented HLA ligands is required to prove visibility of target peptides to T cells. This is a challenge, for example, in the context of cancer immunotherapy approaches that aim to identify optimal tumor-specific HLA-presented antigens.^20 23^ While their discovery has been made possible by proteogenomics approaches, a major impediment still resides in the lack of benign tissues as a reference for the definition of tumor specificity of target peptides.^11 24 25^ Due to the scarce availability of benign human tissue ligandomes, common alternative strategies are based on transcriptomic datasets either from the same patient, or from multiple tissues extracted from publicly available repositories.^3 4^ Frequently, morphologically normal tissue adjacent to the tumor (normal tissues adjacent to tumor, NATs) is used as a control in cancer research. However, NATs have been shown to pose unique challenges, since they may be affected by disease and have been suggested to represent a unique intermediate state between healthy and malignant tissues, with a pan-cancer-induced inflammatory response.^26^ Additionally, for some malignancies, for example, of the brain, surgical resection of NATs is inadmissible. Even in cancers that allow for the extraction of NATs, it is still necessary to investigate the presence of potential tumor-associated antigens (TAAs) on other tissues to anticipate on-target/off-tumor, systemic adverse events when administering immunotherapies to patients.^27 28^

In this study, we, thus, employed tissues originating from research autopsies of subjects that have not been diagnosed with any malignancy and have deceased for other reasons, an approach previously described as a surrogate source of benign tissue.^3 26^ Although these tissues were affected by a range of non-malignant diseases, we designate them as benign to emphasize morphological normality and absence of malignancy. This definition of benign is in agreement with the definition used by the Genotype-Tissue Expression Consortium,^3 4^ which provides RNA sequencing data of benign tissues originating from autopsy specimens.

We performed a large-scale liquid chromatography mass spectrometry (LC-MS/MS)-based characterization of both HLA-I and HLA-II ligands providing data from benign human tissues obtained at autopsy. The HLA Ligand Atlas is a first draft of a pan-tissue immunopeptidomics reference library from benign tissues comprizing for the first time 227 mostly paired HLA-I (198) and HLA-II (220) ligandomes from 29 different benign tissue types obtained from 21 human subjects. For the data analysis, we employed MHCquant,^29^ the first open-source customized computational tool for immunopeptidomics assays that provides database search, false discovery rate (FDR) scoring, label-free quantification and binding affinity predictions. In addition, we implemented a user-friendly, web-based interface to query and access the data at **https://hla-ligand-atlas.org**. Despite its unprecedented comprehensiveness, the HLA Ligand Atlas currently contains only a limited number of tissues and subjects. However, it has been designed as an open and extensible community resource and we invite other researchers to share additional data with us for inclusion in the database. Consistent quality control and data processing will ensure a high quality of the data.

## Results

### The HLA Ligand Atlas: content and scope of the data resource

**Figure 1:**
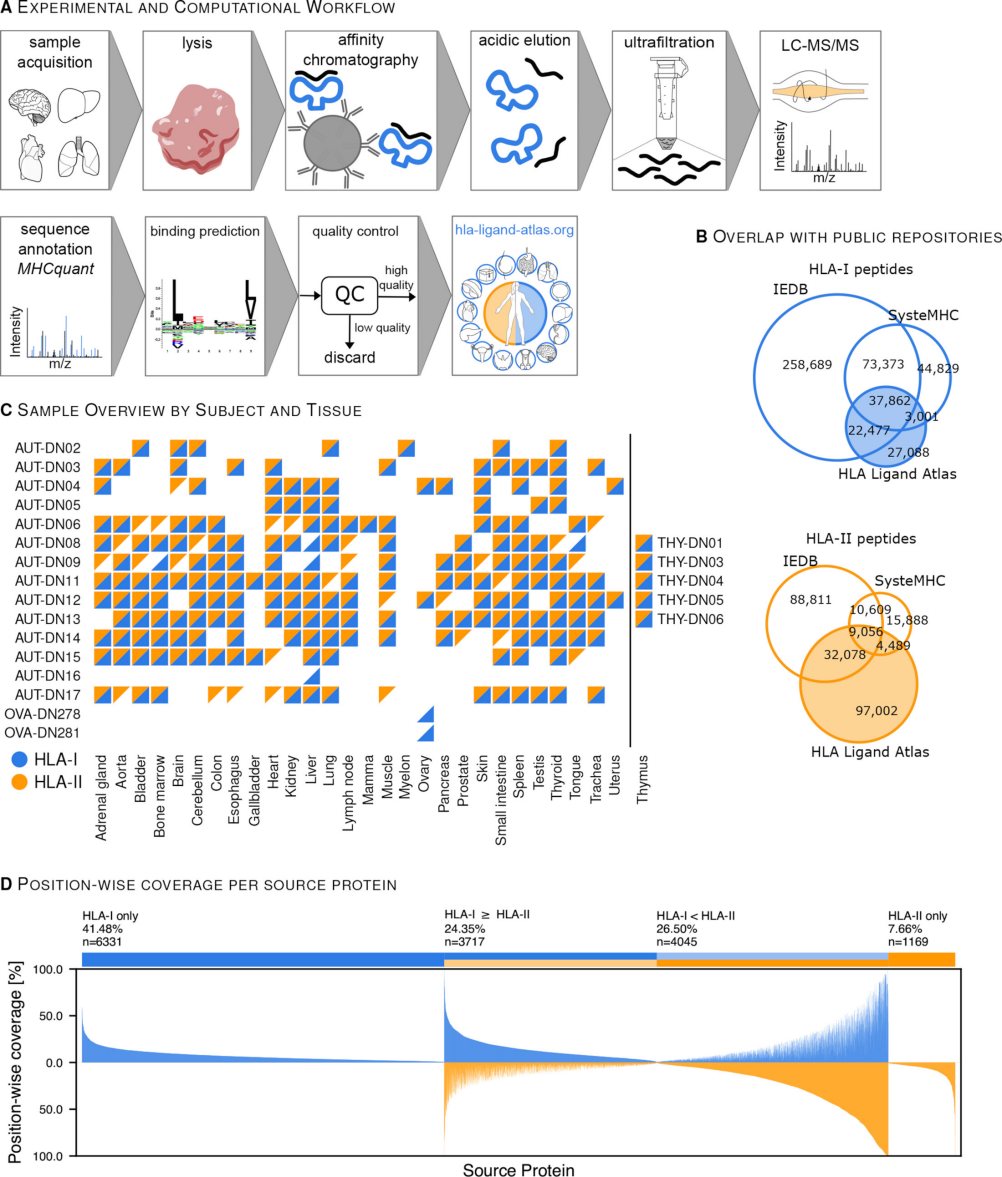
The HLA Ligand Atlas: content and scope of the data resource. (A) The high-throughput experimental and computational workflow steps used to analyze thousands of HLA-I and HLA-II peptides isolated from benign tissues. The resulting HLA-I and HLA-II immunopeptidomes are comprised in the searchable web resource: https://hla-ligand-atlas.org. See online supplemental figure S1 for details of the quality control workflow. See online supplemental figure S2 for proof of principle using autopsy tissues. (B) HLA-I and HLA-II peptides expand the know immunopeptidome as curated in the public repositories SysteMHC and IEDB. (C) Sample matrix: HLA-I- (blue triangles) and HLA-II samples (orange triangles) included in the HLA Ligand Atlas cover 29 different tissues obtained from 21 human subjects. See online supplemental table S1 for patient characteristics. (D) Position-wise coverage (%) of identified source proteins by HLA ligands binned into four groups: (1) exclusively covered by HLA-I peptides, (2) exclusively covered by HLA-II peptides and (3–4) covered by both and separated into higher position-wise coverage by either HLA-I or HLA-II peptides. HLA, human leucocyte antigen; IEDB, immune epitope database; LC-MS/MS, liquid chromatography mass spectrometry.

We describe the HLA Ligand Atlas, a dataset of matched HLA-I and HLA-II ligandomes of benign tissues. HLA-I and HLA-II ligands were isolated via immunoaffinity purification and identified by LC-MS/MS. HLA-binding prediction algorithms and an assessment of peptide length distributions were used to identify high-quality samples and only these were integrated into the dataset (online supplemental figure S1 describes the QC steps employed). Our online resource **https://hla-ligand-atlas.org** provides access to the dataset comprizing HLA-I and HLA-II ligands (1% local peptide-level FDR), their source proteins, tissue and subject of origin, as well as all corresponding HLA allotypes classified as strong or weak binders through several user-friendly views (figure 1A and online supplemental figure S1). We have acquired HLA ligandome data from 29 distinct tissues obtained from 21 individuals, surmounting to 1274 LC-MS/MS runs from 225 mostly paired HLA-I (198) and HLA-II (220) samples (figure 1C and online supplemental table S1. The majority of samples was obtained from 14 subjects after autopsy, while 7 additional subjects contributed 5 thymus and 2 ovary samples after surgery. We performed a time series experiment on three benign samples, two ovaries and one liver (online supplemental figure S2) and observed no qualitative or quantitative degradation of the immunopeptidome for up to 72 hours after tissue removal, supporting the feasibility of employing autopsy tissue as input material for immunopeptidomics assays (online supplemental figure S2).

10.1136/jitc-2020-002071.supp1 Supplementary data

10.1136/jitc-2020-002071.supp2 Supplementary data

Overall, we identified 90,428 HLA-I and 142,625 HLA-II peptides with a local peptide-level FDR of 1% and estimated global peptide-level FDRs of 4.5% and 3.9% for HLA-I and HLA-II peptides, respectively. Identified peptides could be attributed to 51 HLA-I and 81 HLA-II allotypes.

Ultimately, this dataset increases the total number of registered HLA ligands from 440,231 to 467,319 for HLA-I and from 160,931 to 257,933 for HLA-II, as currently encompassed in SysteMHC^30^ and the immune epitope database (IEDB)^31^ (figure 1B).

Moreover, we sought to approximate the worldwide HLA allele frequency of subjects included in the HLA Ligand Atlas. For this purpose, we computed population coverages using the IEDB Analysis Resources (http://tools.iedb.org/population/) (online supplementla table S2). When considering at least one HLA allele match per individual, we observe an allele frequency of 95.1%, 73.6%, 93.0%, for HLA-A (n=16), HLA-B (n=21) and HLA-C (n=14) alleles, respectively. Within the same constraints, we observe allele frequencies of 78.8%, 99.5%, 98.2%, 92.3% for HLA-DPB1 (n=9), HLA-DQA1 (n=11), HLA-DQB1 (n=12) and DRB1 (n=19) alleles, respectively (online supplemental table S2).

10.1136/jitc-2020-002071.supp3 Supplementary data

### Source proteins and HLA allotype coverage characteristics of HLA ligands

**Figure 2:**
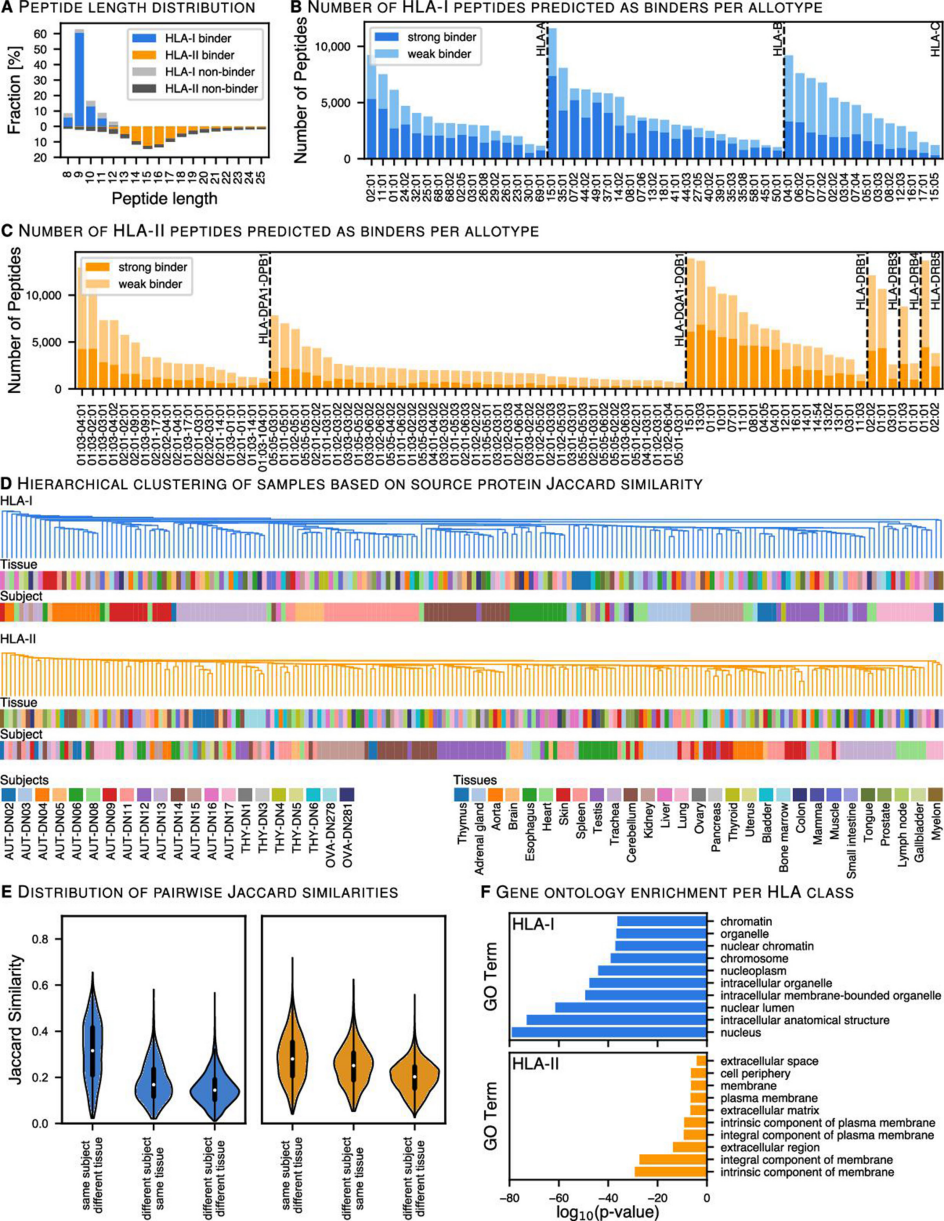
Source proteins and HLA allotype coverage characteristics of HLA ligands. (A) Length distribution of identified HLA-I and HLA-II peptides from all samples was analyzed. HLA-II peptide lengths are mirrored on the negative side of the x-axis. (B, C) Global overview of HLA-I predicted binders distributed across HLA molecules. HLA binding prediction was performed with NetMHCpan-4.1 (% binding rank ≤2) and SYFPEITHI (Score >50%), while multiple HLA allotypes per peptide were allowed as long as their scores met the aforementioned thresholds. HLA binding prediction for HLA-II ligands was performed with NetMHCIIpan-4.0 and MixMHC2pred (% binding rank ≤5 for both). (D) Pairwise hierarchical clustering of samples based on the Jaccard similarity between HLA-I (blue) and HLA-II (orange) source proteins. The dendrogram illustrates the nearest neighbor based on the similarity between tissues and subjects. (E) Violin plots illustrate the distribution of the Jaccard similarity index for each pairwise comparison between the same subject—different tissues; different subjects—the same tissue and different subject—different tissues. (F) Gene ontology (GO) term enrichment of cellular components was performed for HLA-I and HLA-II source proteins. Top10 enriched genes with respect to their log_10_ p value (Fisher’s exact test) differentiate between intracellular and extracellular antigen processing pathways. HLA, human leucocyte antigen.

The HLA ligands in the dataset were identified based on 15,262 of the 20,365 proteins in Swiss-Prot, hereinafter referred to as source proteins. About half of these source proteins yield both HLA-I and HLA-II ligand identifications, 40% yield only HLA-I ligands and 8% only HLA-II ligands (figure 1D). We performed a gene ontology (GO) enrichment analysis of HLA-I and HLA-II exclusive source proteins, which corroborates the expected cellular compartments associated with the class-specific antigen presentation pathways, with HLA-I presenting primarily intracellular and HLA-II primarily extracellular proteins (figure 2F).

When looking at single amino acid residues across all source proteins (position-wise), 10% of the single residue positions are covered by HLA ligands, a parameter that ranges from 0.02% to 1.15% for individual HLA allotypes (online supplemental figure S3). The mode of the overall peptide length distribution depicts the highest abundance of 9mers (60%) for HLA-I and of 15mers (18%) for HLA-II ligands (figure 2A). While 82% of the HLA-I ligands are predicted to bind a subject’s HLA allotype, this holds true for only 62% of the HLA-II ligands. A major shortcoming of HLA-II binding prediction models appears to be a negative bias toward the tails of the observed peptide length distribution, in particular toward short peptides (figure 2A). The number of identified peptides that are predicted to bind to specific allotypes varies strongly between allotypes, with HLA-A*02:01, HLA-B*15:01, HLA-B*35:01, HLA-C*04:01 and most HLA-DRB1 allotypes being among the highly represented ones (figure 2B, C).

### The interindividual heterogeneity outweighs similarities between tissue types

An unaddressed question, relevant for the discovery and administration of shared TAAs, is whether the similarity between tissue types outweighs that between individuals. We assessed the similarity of the immunopeptidome on both source protein (figure 2D, E) and HLA-ligand level (online supplemental figure S4C, D) between samples, as defined by subject-tissue combinations. For this purpose, we computed pairwise similarities between all samples as measured by the Jaccard similarity index and clustered the samples based on their similarity.

We observe that even on the source protein level there is a higher similarity between samples that originate from the same subject over samples that originate from the same tissues. Notably, while this effect is to some degree expected due to the allotype specific presentation behavior, it persists when only allele matched samples and peptides are taken into account (online supplemental figure S5). Prominent exceptions are the thymus samples, which form clusters within the HLA-I and HLA-II samples despite originating from different donors.

### The immunopeptidome yield varies consistently across tissues

**Figure 3:**
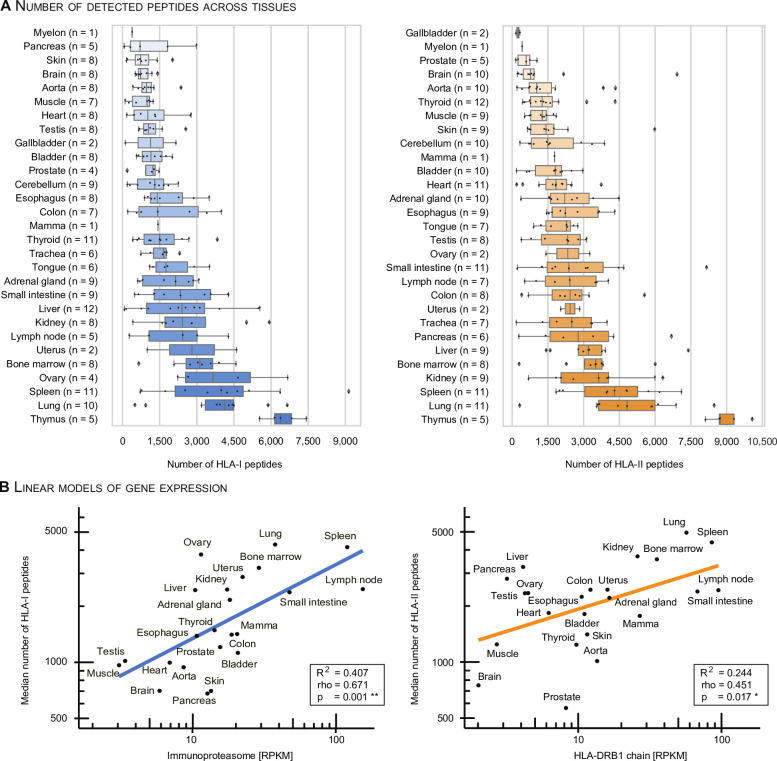
Tissues exhibit a gradual separation based on the immunopeptidome yield. (A) The number of identified HLA-I and HLA-II peptides per sample (subject and tissue combinations) was sorted and plotted by median immunopeptidome yield per tissue. Boxes span the inner two quantiles of the distribution and whiskers extend by the same length outside the box. Remaining outlier samples are indicated as black diamonds. The number of subjects contributing to each tissue is illustrated on the y-axis in parenthesis. (B) A linear model was used to correlate the log transformed HLA-I and HLA-II median peptide yields with log transformed median gene expression counts (RPKM) of the immunoproteasome and HLA-DRB1 per tissue^32^. Corresponding R^2^, p value (F-statistic) and spearman rho are indicated in the bottom right box. HLA, human leucocyte antigen.

We observe a strong variance in the immunopeptidome yield, defined as the number of identified peptides per sample, across all tissues (figure 3A) and subjects (online supplemental figure S4A, B). Despite the interindividual (i.e. inter-allotype) variance, we can consistently differentiate between high-yielding and low-yielding tissues with respect to both HLA-I and HLA-II peptides (figure 3 and online supplemental figure S4A, B). The separation of tissues based on the immunopeptidome yield is not abrupt, but gradual. Low-yielding tissues include skin, aorta, brain and the gallbladder with a low number of both HLA-I and HLA-II presented peptides across all subjects. On the other hand, high-yielding tissues include thymus, lung, spleen, bone marrow and kidney (figure 3A). These tissues have well-characterized immune-related functions or are central components of the lymphatic system.

We employed a linear model to systematically evaluate the correlation between the median HLA-I/HLA-II immunopeptidome yield with RNA expression values (reads per kilobase per million - RPKM) of immune-related genes identified by targeted RNA sequencing from an external dataset^32^ (figure 3B and online supplemental figure S6). We observe a significant correlation between expression values of immune-related genes and HLA-I and HLA-II immunopeptidome yields (online supplemental figure S6). Among these, genes of the immunoproteasome correlate well with the number of HLA-I ligand identifications per tissue (R^2^=0.407, rho=0.671, p=0.001, figure 3B, left). Independent studies mapping the healthy human proteome confirm expression of the immunoproteasome in a wide range of tissues, including tissues for which no primary immunological function would be expected.^6 7^

10.1136/jitc-2020-002071.supp6 Supplementary data

HLA-II peptide yields correlate well with the expression of HLA-DRB1 genes (R^2^=0.244, rho=0.451, p=0.017, figure 3B, right). HLA-DR is well characterized due to the invariant α chain, and thus reduced complexity in the peptide binding groove. Through the high specificity of the L243 antibody for HLA-DR, and the presumably varying specificity of the second antibody Tü39 for different HLA-II allotypes, we cannot exclude a skewed identification in favor of HLA-DRB allotypes. However, higher expression values for HLA-DRB1 compared with other HLA-II allotypes have been described for example in earlier studies on gastric epithelium.^33^

### Small subsets of source proteins are tissue exclusive

**Figure 4:**
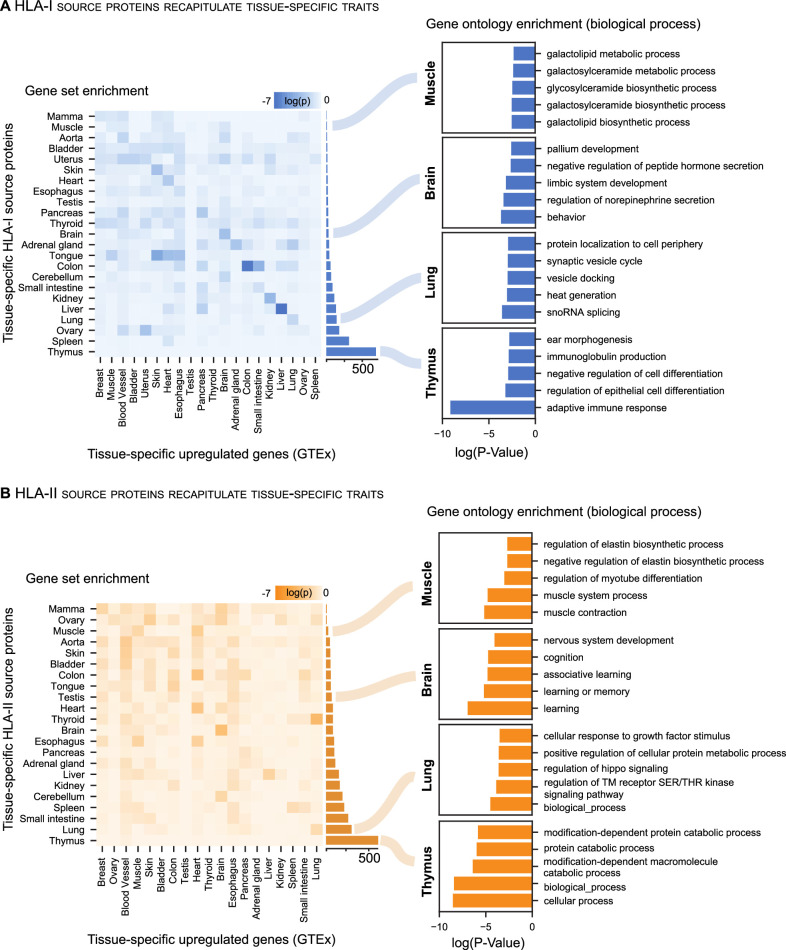
Small subsets of source proteins are tissue exclusive. (A, B) Gene set enrichment (left) was tested for each tissue by correlating unique HLA-I and HLA-II source proteins per tissue with upregulated genes as annotated in GTEx. Heatmaps depict log_10_ p values (Fisher’s exact test) for each pairwise comparison. The number of tissue-specific HLA-I and HLA-II source proteins is depicted through the bar plot for each tissue on the right-hand side of the heatmaps. In addition, GO term enrichment (right) of biological processes was performed using the panther DB webservice for selected tissues with the same set of HLA-I and HLA-II tissue-specific source proteins. Top five enriched terms with respect to their log_10_ p value (Fisher’s exact test) were selected. DB, database; GO, gene ontology; HLA, human leucocyte antigen.

Previous studies characterizing the human transcriptome and proteome across tissues have shown varying degrees of tissue specificity for transcripts and proteins.^34 35^ In this context, we analyzed source proteins of the benign immunopeptidome as a whole and grouped all samples by tissue of origin. We observe a particularly small number of HLA-I (ranging from 5 in mamma to 674 in thymus), and HLA-II (ranging from 8 in ovary to 564 in thymus) source proteins identified exclusively in one tissue (figure 4A, B and online supplemental table S5). Concordantly, only small numbers of tissue-exclusive protein identifications have been observed in human tissue-wide proteomics studies.^6^ Only recently, the systematic, quantitative analysis of the human proteome and transcriptome in multiple tissues has revealed that differences between tissues are rather quantitative than defined by the presence or absence of certain proteins.^34 35^

10.1136/jitc-2020-002071.supp4 Supplementary data

To determine whether tissue-specific biology is conserved between the transcriptome and immunopeptidome, we compared tissue-enriched gene sets from the GTEx repository with tissue-exclusive HLA-I and HLA-II source proteins (figure 4A, B, left). We observe that tissue-specific biology is represented by HLA-I and HLA-II source proteins through an enrichment with upregulated transcripts in the respective tissue.

We additionally observed that tissue-specific traits are recapitulated by GO term enrichment of biological processes (figure 4A, B, right). Enriched GO terms reveal tissue-specific biological functions such as ‘adaptive immune response’ in the thymus or ‘behavior’ in the brain. However, clear associations between enriched gene sets and HLA-I and HLA-II source proteins are less evident in tissues such as spleen or testis, despite the disparity of tissue-exclusive HLA-I source protein identifications, accounting for only 23 in testis, while spleen yields 308.

Overall, tissue-specific traits are more evident for HLA-I than for HLA-II source proteins when assessing the correlation between tissue-exclusive source proteins with GTEx-enriched transcripts and function-specific GO terms. HLA-II source proteins are represented by more general GO terms, which still reflect distinct biological processes characteristic for the respective tissue.

### Cryptic peptides are part of the benign immunopeptidome

**Figure 5:**
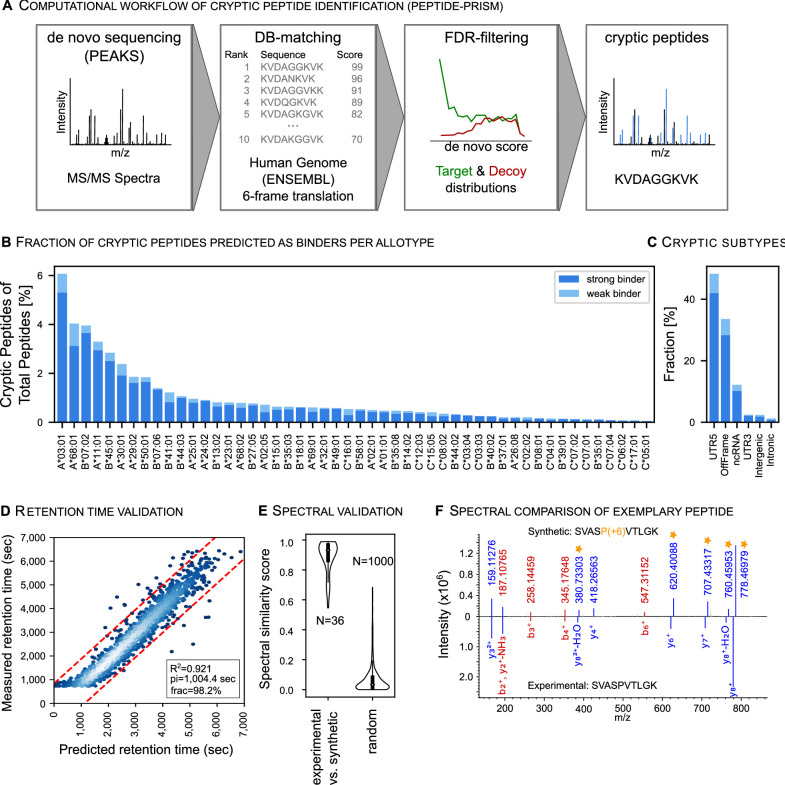
Cryptic peptides are part of the benign immunopeptidomes. (A) Spectra were searched with Peptide-PRISM to identify peptides of cryptic origin. Briefly, *de novo* sequencing was performed, and top 10 sequences per spectrum were queried against a database consisting of the three-frame translated transcriptome (Ensemble 90) and the six-frame translated human genome (HG38). Target-Decoy search was performed per database stratum, separately for canonical and cryptic peptides. (B) The HLA-allotype distribution of cryptic peptides was plotted in relation to cryptic and canonical peptides predicted to bind to the respective HLA allotype across all subjects and tissues. (C) Distribution of identified cryptic peptides categorized into multiple non-coding genomic regions. (D) Linear model correlating measured retention times (RT) of cryptic peptides with their predicted RTs trained on canonical peptide RTs. Corresponding R^2^, pi (width of the prediction interval—red dashed lines) and frac (the number of peptides falling into the prediction interval) are indicated in the bottom right. (E) 36 cryptic peptides were selected for spectral validation with synthetic peptides. The similarity between the synthetic and experimental spectrum was computed by correlation scores. (F) Exemplary spectral comparison of the cryptic peptide SVASPVTLGK and its synthesized heavy isotope-labeled counterpart (P+6). Matching b (red) and y ions (blue) are indicated as well as the isotope mass shifted ions (orange stars) of the synthesized peptide. FDR, false discovery rate; HLA, human leucocyte antigen.

Recently, cryptic HLA peptides came into focus as a new potential source of TAAs. Cryptic peptides originate from non-coding regions, that is, 5’- and 3’- untranslated region (UTR), non-coding RNAs (ncRNA), intronic and intergenic regions, or from shifted reading frames in annotated protein coding regions (off-frame). Ribosome profiling and immunopeptidomics studies confirm their translation and presentation on HLA-I molecules.^24 25 36^ So far, cryptic peptides have predominantly been characterized in tumors, while their presentation in benign tissues remains poorly charted. We analyzed the HLA-I-restricted LC-MS/MS data of the HLA Ligand Atlas with Peptide-PRISM^37^ (figure 5A) and identified 1407 cryptic peptides, including the peptide SVASPVTLGK that was classified as a TAA in lung cancer tissue in a previously published study (figure 5F and online supplemental table S3).^25^ This peptide was identified in the HLA Ligand Atlas in five different subjects in lung and liver tissues. We find that 47% of cryptic peptides were identified in more than one subject (online supplemental table S3). Both cryptic and conventional peptides share similar physicochemical properties. Their predicted chromatographic retention time correlates with their experimentally observed retention time equally well as for conventional peptides (figure 5D).^24 36^ The identified cryptic peptides predominantly originate from 5’-UTRs (figure 5C), which is in accordance with previous studies.^36 37^ Overall, HLA allotypes show different presentation propensities of cryptic peptides, when related to cryptic and canonical peptides, with HLA-A*03:01 covering the largest fraction of all identified cryptic peptides, followed by HLA-A*68:01 and HLA-B*07:02, as previously observed (figure 5B).^37^

10.1136/jitc-2020-002071.supp5 Supplementary data

We selected 36 top-ranking (1% FDR) cryptic peptides, shared among subjects for spectral validation by experimental comparison with the corresponding isotopically-labeled synthetic peptide (online supplemental table S3). We were able to confirm the correct identification of selected cryptic HLA-I ligands by comparing the pairwise similarity (spectral angle) between experimental and synthetic peptides against a random distribution (figure 5E) as well as through individual inspection (online supplemental figure S7). In summary, we can show that cryptic peptides are not per-se tumor-specific, although their frequency might be reduced in benign tissues.^37^

### HLA Ligand Atlas data enables prioritization of TAAs

**Figure 6:**
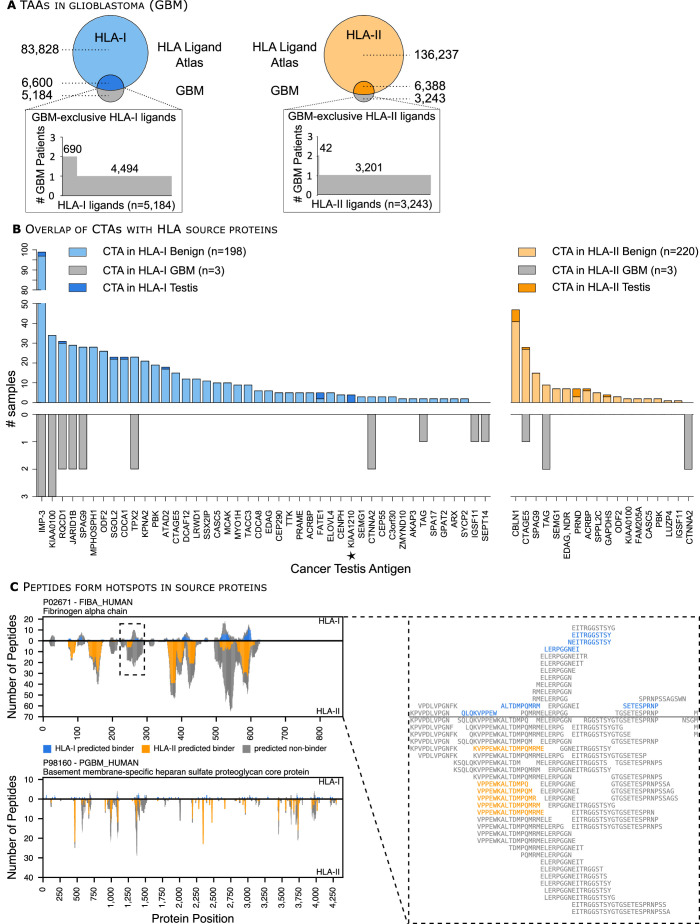
HLA Ligand Atlas data enables prioritization of tumor-associated antigens (TAAs) and HLA ligands form hotspots in source proteins. (A) The size-proportional Venn diagram illustrates the overlap between the pooled glioblastoma (GBM) and benign HLA-I and -II immunopeptidomes, respectively. The waterfall plots show the number of glioblastoma-associated HLA-I ligands and their frequency among the three GBM patients analyzed. (B) Published CTAs are presented as HLA-I or HLA-II ligands on benign tissues, including testis but also in glioblastoma tumors. The number of identified samples either from the HLA Ligand Atlas or the glioblastoma dataset is depicted on the x-axis, provided that each CTA has been identified with at least two different HLA ligands. The CTA KIA1210 was identified exclusively on HLA-I source proteins in testis and is marked with an asterisk. (C) The position-wise HLA ligand coverage profiles as available in the HLA Ligand Atlas web interface for two exemplary proteins (left), the fibrinogen alpha chain (Uniprot ID P02671, length 866 aa, top) and the basement membrane-specific heparan sulfate proteoglycan core protein (Uniprot ID P98160, length 4391 aa, bottom) are shown, illustrating the spatial clustering of HLA ligands into hotspots. For P02671 a close-up of such a cluster is shown in form of a multiple sequence alignment of the identified peptides (right). CTAs, cancer testis antigens; HLA, human leucocyte antigen, GBM, glioblastoma.

On-target/off-tumor adverse events in a clinical immunotherapeutic setting can have fatal consequences.^27 28^ To minimize the risk of on-target/off-tumor adverse events, multi-tissue immunopeptidomics reference libraries from benign tissues are required to identify TAAs.^24 25^ Here, we propose the implementation of the HLA Ligand Atlas as a reference library of benign multi-tissue immunopeptidomes for comparative profiling with tumor immunopeptidomes for the discovery of actionable TAAs. As a case study, we selected three glioblastoma tumor samples from different individuals and analyzed their immunopeptidomes. We comparatively profiled the 11,784 HLA-I and 9631 HLA-II ligands identified in the glioblastoma samples against the benign dataset encompassed in the HLA Ligand Atlas (figure 6A, B). The majority of HLA ligands is shared between both tumor and benign tissues, with 5185 HLA-I TAAs and 3243 HLA-II TAAs being unique to glioblastoma (online supplemental table S4). When assessing their presentation frequency, 690 HLA-I TAAs are found on two glioblastoma samples, while 4494 are patient-individual. In the case of HLA-II TAAs, 42 are shared between two glioblastoma patients, and 3201 are patient individual. No identified HLA-I or HLA-II ligands were common to all three glioblastoma patients.

Moreover, we investigated the presentation of cancer testis antigens (CTAs) by HLA-I and HLA-II molecules on benign tissues. CTAs are immunogenic proteins preferentially expressed in normal gametogenic tissues and different types of tumors. We compiled a list of 422 published CTAs from the curated CT database^38^ and a recent publication aiming to identify CTAs from transcriptomics datasets.^39^ Of 422 published CTAs, 49 CTAs were presented on either HLA-I or HLA-II molecules and 10 CTAs on both HLA-I and HLA-II molecules in the HLA Ligand Atlas, provided that respective source proteins were identified with at least two HLA ligands (figure 6B and online supplemental table S4). CTAs, such as CTAGE5, KIA0100 and SPAG9, were presented in numerous benign samples with HLA-I and HLA-II ligands (figure 6B and online supplemental table S4). Furthermore, the CTA KIA1210 was only identified in the benign dataset on testis in accordance to its CTA status. Similarly, we queried all glioblastoma source proteins against the selected 422 CTAs and found three CTAs (two HLA-I and one HLA-II) exclusively presented in glioblastoma and not in our benign dataset, indicating promising targets against this tumor entity.

### HLA ligands form hotspots in source proteins

When looking at the position-wise coverage profiles of individual source proteins across all HLA allotypes, we observe that HLA ligands seem to emerge from spatially clustered hotspot regions while other areas of the source protein do not contribute any HLA ligands at all (figure 6C, left). It has been shown previously that this clustering effect cannot be explained by the occurrence of HLA binding motifs as incorporated in epitope prediction tools.^40^ The hotspot locations often coincide between HLA-I and HLA-II ligands, however, we did not perform a large-scale statistical analysis to validate this class linkage effect. In the case of HLA-II, the clustering effect has to be distinguished from the co-occurrence of HLA-II ligand length variants, which leads to a large number of distinct peptides covering the same source protein position due to the nature of HLA-II antigen processing and binding.^41^ Many of the observed clusters span ranges of distinct, non-overlapping HLA-II ligands (figure 6C, right). Position-wise coverage plots for all source proteins are available online at http://hla-ligand-atlas.org.

### The HLA Ligand Atlas web interface

The HLA Ligand Atlas web interface was designed to allow users to conveniently access the data we collected. Users can formulate queries in the form of filters based on peptide sequences, peptide sequence patterns, HLA allotypes, tissues and proteins of origin, or combinations thereof. Additionally, users can submit files with peptides or UniPort IDs, either as plain lists or as a FASTA files. The peptide list is then queried against the database and the resulting hits can again be freely combined with the aforementioned filters. Query results are shown as a list of peptides with plots of the corresponding HLA allotype and tissue distributions. Additionally, detailed views for single peptides and for coverage of proteins are available. Apart from the query interface, the web front-end also displays various aggregate views of the data stored in the database.

## Discussion

The HLA Ligand Atlas provides for the first time a comprehensive collection of benign human HLA-I and HLA-II immunopeptidomes. Both the experimental and computational workflows were designed in a standardized and reproducible fashion. The data resource enables further data-driven research to improve our mechanistic understanding of antigen presentation and will substantially enhance HLA binding prediction models. In addition, the analyses presented here already underline the individuality of HLA ligandomes, even beyond the rather well understood heterogeneity imposed by HLA allotypes. Our evaluation of pairwise sample similarity provides evidence that differences between individuals surpass differences between tissue types in the same individual for both the immunopeptidome and their source proteins even when samples are matched for HLA allotypes. On the proteome^34^ and transcript levels,^3 4^ however, samples were previously shown to cluster by tissue type, rather than by individual. In line with that finding, a weak correlation between immunopeptidome yield and RNA expression values has been observed previously.^10 11^ It is evident that allotype-specific presentation by HLA is crucial to explain the different behavior on the level of proteomes and ligandomes. However, when comparing HLA-matched datasets, the clustering by individual is still evident and thus additional effects (eg, other—potentially unknown—steps in the HLA-I antigen processing pathway) could play an important role as well.

This high degree of individuality between immunopeptidomes has major repercussions for clinical applications in emerging fields such as immuno-oncology. Our findings indicate that the immunopeptidome adds an additional layer of complexity to the well-described genomic and transcriptomic tumor-heterogeneity. Successful induction of T-cell responses after peptide vaccination with neoantigens^42 43^ indicate that precision medicine will evolve to an increasingly individualized field, where treatment options will be tailored to the immunopeptidomic landscape of the tumor. Mapping the tumor HLA ligandome of an individual patient therefore needs to be paralleled by a broad and in-depth knowledge of its benign counterpart—the HLA Ligand Atlas is a first step in this direction.

An essential application of the HLA Ligand Atlas is the selection of candidate peptide targets for immunotherapy approaches. We propose to prioritize the large candidate pool of non-mutated tumor-associated targets by comparatively profiling immunopeptidomes of primary tumors and benign tissues, as provided by the HLA Ligand Atlas. This approach would complement current strategies based on transcriptomes of benign tissues as comprised for example in GTEx.^3 4^ The HLA Ligand Atlas represents a first draft of a tissue-wide immunopeptidomics map covering both HLA-I and HLA-II canonical peptides, but also HLA-I non-canonical peptides that can be employed as an orthogonal level of quality control when defining TAAs.

Advances in LC-MS/MS technology, data acquisition methods and computational tools are constantly improving the depth of coverage of immunopeptidomics experiments. Therefore, we encourage the reanalysis of the raw LC-MS/MS dataset with novel hypotheses and upcoming computational methods that will lead to additional insight. Overall, we anticipate that the number of charted human immunopeptidomes will increase, similarly as the human genome and transcriptome were mapped across multiple individuals. By generating larger datasets from many human individuals, population-wide conclusions can be drawn, and immunopeptidome-wide studies will provide insight into disease-associated HLA alleles and peptides.^14^ The HLA Ligand Atlas strives to advance our understanding of fundamental aspects of immunology relating to autoimmunity, infection, transplantation, cancer immunotherapy and might provide a foundation for vaccine design. We hope that together with the scientific community we can expand the benign immunopeptidome to encompass more human subjects, tissues and HLA alleles.

## Methods

### Experimental model and subject details

Human tissue samples were obtained post-mortem during autopsy performed for medical reasons at the University Hospital Zürich. None of the subjects included in this study was diagnosed with any malignant disease. Tissue samples were collected during autopsy, which was performed within 72 hours after death. Tissue annotation was performed by a board-certified pathologist. Tissue samples were immediately snap-frozen in liquid nitrogen.

Thymus samples were obtained from the University Children's Hospital Zürich/ Switzerland. Thymus tissue was removed during heart surgery for other medical reasons.

Furthermore, two benign ovarian tissue samples were collected for the time series experiments (OVA-DN278 and OVA-DN281). Both patients were post-menopausal and had a bilateral ovarectomy for cystadenofibromas, which were diagnosed by histopathological examination of the specimen. The samples were obtained from a normal part of the ovary.

Finally, we included three primary glioblastoma tumor samples to illustrate a selection strategy for TAAs. The primary glioblastoma tumor was analyzed for patients GBM616 and GBM654, whereas, a recurrent tumor was analyzed for GBM753.

### HLA typing

Multiple HLA typing approaches were performed for the different sources of patient material.

Autopsy subject AUT-DN08, AUT-DN16, and two benign ovary samples (OVA-DN278 and OVA-DN281) were typed at the Department of Transfusion Medicine of the University Hospital of Tübingen. High-resolution HLA typing was performed by next-generation sequencing on a GS Junior Sequencer using the GS GType HLA Primer Sets (both Roche, Basel, Switzerland). HLA typing was successful for HLA-A, -B, and -C alleles. However, HLA-II typing was only reliable for the HLA-DR locus, and incomplete for the HLA-DP and -DQ loci.

Therefore, we performed exome sequencing of lung tissue for remaining autopsy subjects. The HLA-I and HLA-II alleles were identified from the exome sequencing data using an improved version of OptiType^44^ available online at https://github.com/FRED-2/OptiType (tagged hla-ligand-atlas).

Finally, sequence-based typing was performed for the five thymus samples and the three glioblastoma samples, by sequencing exons 1–8 for HLA-I alleles and exons 2–6 for HLA-II alleles (Histogenetics, Ossining, New York, USA).

The subject characteristics are summarized in online supplemental table S1 encompassing information on sex, age, the number of collected tissues and HLA-I and HLA-II alleles.

### HLA immunoaffinity purification

HLA-I and HLA-II molecules were isolated from snap-frozen tissue using standard immunoaffinity chromatography. The antibodies employed were the pan-HLA-I-specific antibody W6/32,^45^ and the HLA-DR-specific antibody L243,^46^ produced in house (University of Tübingen, Department of Immunology) from HB-95, and HB-55 cells (ATCC, Manassas, Virginia, USA), respectively. Furthermore, the pan-HLA-II-specific antibody Tü39 was employed and produced in house from a hybridoma clone as previously described.^47^ The antibodies were cross-linked to CNBr-activated sepharose (Sigma-Aldrich, St. Louis, Missouri, USA) at a ratio of 40 mg sepharose to 1 mg antibody for 1 g tissue with 0.5 M NaCl, 0.1 M NaHCO_3_ at pH 8.3. Free activated CNBr reaction sites were blocked with 0.2 M glycine.

For the purification of HLA-peptide complexes, tissue was minced with a scalpel and further homogenized with the Potter-Elvehjem instrument (VWR, Darmstadt, Germany). The amount of tissue employed for each purification is documented in online supplemental table S1. This information is not available for seven tissues, annotated as n.d. in said table. Tissue homogenization was performed in lysis buffer consisting of CHAPS (Panreac AppliChem, Darmstadt, Germany) and one cOmplete protease inhibitor cocktail tablet (Roche) in PBS. Thereafter, the lysate was sonicated and cleared by centrifugation for 45 min at 4000 rpm, interspaced by 1-hour incubation periods on a shaker at 4°C. Lysates were further cleared by sterile filtration employing a 5 µm filter unit (Merck Millipore, Darmstadt, Germany). The first column contained 1 mg of W6/32 antibody coupled to sepharose, whereas the second column contained equal amounts of Tü39 and L243 antibody coupled to sepharose. Finally, the lysates were passed through two columns cyclically overnight at 4°C. Affinity columns were then washed for 30 min with PBS and for 1 hour with water. Elution of peptides was achieved by incubating four times successively with 100–200 µL 0.2% trifluoroacetic acid (TFA) on a shaker. All eluted fractions were subsequently pooled. Peptides were separated from the HLA molecule remnants by ultrafiltration employing 3 kDa and 10 kDa Amicon filter units (Merck Millipore) for HLA-I and HLA-II, respectively. The eluate volume was then reduced to approximately 50 µL by lyophilization or vacuum centrifugation. Finally, the reduced peptide solution was purified five times using ZipTip pipette tips with C18 resin and 0.6 µL bed volume (Merck,) and eluted in 32.5% acetonitrile (ACN)/0.2% TFA. Each peptide eluate was purified by loading it five times onto the same ZipTip pipette tip. The tip was passed sequentially by pipetting ten times up and down through 32.5% ACN/ 0.2% TFA for purification, 0.1% TFA for equilibration, the sample for binding the peptides, 0.1% TFA for desalting and 32.5% ACN/0.2% TFA for elution. This entire sequence was repeated five times using the same ZipTip pipette tip. The purified peptide solution was concentrated by vacuum centrifugation and supplemented with 1% ACN/0.05% TFA and stored at −80°C until LC-MS/MS analysis.

### Time series experiments

We performed time series experiments to assess the suitability of tissues obtained from autopsies as a source of human tissues for the characterization of the benign immunopeptidome. We evaluated the degradation profile of the immunopeptidome, when tissues were stored at 4°C for up to 72 hours after tissue removal, to mimic the conditions at autopsy. The time series experiment was repeated in three benign tissues from different individuals: one benign liver obtained at autopsy (AUT-DN16 Liver), and two benign ovaries removed surgically (OVA-DN278 and OVA-DN281). The tissues were extracted and incubated at 4°C until the defined time point and flash-frozen in liquid nitrogen until HLA ligand extraction. As more tissue was available form AUT-DN16 Liver, tissue samples were frozen after 8 hours, 16 hours, 24 hours, 48 hours and 72 hours. Due to the limited sample amount obtained from OVA-DN278 and OVA-DN281, only three time points could be accounted for: 0 hours, 24 hours and 72 hours. The HLA immunoaffinity purification was performed as mentioned, with the exception that mass to volume ratio in ovary samples was adjusted to the lowest mass across all time points before loading onto sepharose columns.

### Mass spectrometric data acquisition

HLA ligand characterization was performed on an Orbitrap Fusion Lumos mass spectrometer (Thermo Fisher Scientific, San Jose, California, USA) equipped with a Nanospray Flex Ion Source (Thermo Fisher Scientific) coupled to an Ultimate 3000 RSLC Nano UHPLC System (Thermo Fisher Scientific). Peptide samples were loaded with 1% ACN/ 0.05% TFA on a 75 µm x 2 cm Acclaim PepMap 100 C18 Nanotrap column (Thermo Fisher Scientific) at a flow rate of 4 µL/min for 10 min. Separation was performed on a 50 µm × 25 cm PepMap RSLC C18 (Thermo Fisher Scientific) column, with a particle size of 2 µm. Samples were eluted with a linear gradient from 3% to 40% solvent B (80 %/0.15% FA in water) at a flow rate of 0.3 µL/min over 90 min. The column was subsequently washed by increasing to 95% B within 1 min, and maintaining the gradient for 5 min, followed by reduction to 3% B and equilibration for 23 min.

Data acquisition was performed as technical triplicates in data-dependent mode, with customized top speed (3 s) methods for HLA-I- and HLA-II-eluted peptides. HLA-I peptides have a length of 8–12 amino acids,^48 49^ therefore, the scan range was restricted to 400–650 m/z and charge states of 2–3. MS1 and MS2 spectra were detected in the Orbitrap with a resolution of 120,000 and 30,000, respectively. Furthermore, we set the automatic gain control (AGC) targets to 1.5*10^5^ and 7.0*10^4^ and the maximum injection time to 50 ms and 150 ms for MS1 and MS2, respectively. The dynamic exclusion was set to 7 s. Peptides were fragmented with collision-induced dissociation while the collision energy was set to 35%.

HLA-II peptides have a length of 8–25 amino acids,^49 50^ thus, the scan range was set to 400–1000 m/z and the charge states were restricted to 2–5. Readout for both MS1 and MS2 were performed in the Orbitrap with the same resolution and maximum injection times as for HLA-I peptides. The dynamic exclusion was set to 10 s and AGC values employed were 5.0*10^5^ and 7.0*10^4^ for MS1 and MS2, respectively. Higher-energy collisional dissociation fragmentation with 30% collision energy was employed for HLA-II peptides.

The LC-MS/MS immunopeptidomics data comprised in the HLA Ligand Atlas has been deposited to the ProteomeXchange Consortium via the PRIDE 51partner repository.

### Database search with MHCquant

MS data obtained from HLA ligand extracts was analyzed using the nf-core^52^ containerized, computational pipeline MHCquant^29^ (release V.1.5.1 - https://www.openms.de/mhcquant/) with default settings. The workflow comprises tools to analyze LC-MS/MS data of the open-source software library OpenMS (V.2.5).^53^ Identification and post-scoring were performed using the OpenMS adapters to Comet 2016.01 rev.3^54^ and Percolator V.3.4^55^ at a local peptide-level FDR threshold of 1% among the technical replicates per sample. Subsequently, we estimated the global peptide-level FDR by dividing the sum of expected false positive identifications from each sample (1% peptide level FDR) by the total number of identified peptides in the entire dataset (HLA-I: 4.5% FDR, HLA-II: 3.9% FDR).^56 57^ The human reference proteome (Swiss-Prot, Proteome ID UP000005640, 20,365 protein sequences) was used as a database reference. Database search was performed without enzymatic restriction, with methionine oxidation as the only variable modification. MHCquant settings for high-resolution instruments involving a precursor mass tolerance of 5 ppm and a fragment bin tolerance of 0.02 Da were applied. The peptide length restriction, digest mass and charge state range were set to 8–12 amino acids, 800–2500 Da and 2–3 for HLA-I and 8–25 amino acids, 800–5000 Da and 2–5 for HLA-II, respectively. No protein inference was performed, however, all proteins that contain a given peptide were annotated as peptide source proteins.

### HLA binding prediction

Peptide binding predictions were computed based on the subject’s HLA alleles. For HLA-I ligand extracts, we employed SYFPEITHI^58^ and NetMHCpan-4.1^59^ in ligand mode (default). The SYFPEITHI score $s_{\text{SYF}}$ was computed by dividing the sum of amino acid-specific values for each position in the tested peptide by the maximally attainable score for the respective HLA allotypes.^60^ HLA-II ligand extracts were annotated with NetMHCIIpan-4.0^18^ and MixMHC2pred^17^ using the default settings.

Peptides were categorized as strong binders against a given HLA allotype if any tool reported it as such (netMHCpan-4.1 $s_{\text{rank}}\leq 0.5$, netMHCIIpan-4.0 $s_{\text{rank}}\leq 1.0,$ MixMHC2pred $s_{\text{rank}}\leq 1.0$, where $s_{\text{rank}}$ is the reported percentile rank score). Peptides were otherwise reported as weak binders if any of the tools reported it as such (netMHCpan-4.1 $s_{\text{rank}}\leq 2.0$, netMHCIIpan-4.0 $s_{\text{rank}}\leq 5.0,$ MixMHC2pred $s_{\text{rank}}\leq 5.0$, SYFPEITHI $s_{\text{SYF}}\geq 0.5$). All peptide-HLA allotype associations within these limits were included in the dataset, that is, a single peptide sequence can be reported as a binder against multiple allotypes of the same donor. Throughout this article, unless allele associations are specified, all peptides including those classified as non-binders against any subject’s allotype were included in the analysis.

### Binding prediction and length distribution-based quality control

We defined the fraction of predicted binders of a sample as the ratio of predicted binders divided by the total number of peptide identifications. Technical replicates with a fraction of predicted binders lower than 50% for HLA-I and lower than 10% for HLA-II ligand extracts were excluded from the dataset. Furthermore, individual replicates were removed from the dataset if the mode of the length distribution differed from nine amino acids for HLA-I and was not in the interval [12, 18] for HLA-II (see online supplemental file 1).

### Quantitative time series analysis

Database search of LC-MS/MS data from the three time series experiments was performed with MHCquant V.1.5.1 as previously described.^29^ Identifications were matched between runs^61^ based on retention time alignment and targeted feature extraction^62^ to integrate respective MS1 areas for all time points and technical replicates.

MS1 areas x were normalized to z-scores (standard scores) z per MS run by subtracting the mean and dividing by SD:

$$z=\frac{\left(x-\mu \right)}{\sigma }$$

The trajectory of scaled MS1 areas was clustered by k-means unsupervised clustering with six seeds using the tslearn (V.0.3.1) python package. All trajectories are related to the first time point by subtracting its median z-score from all other timepoints in the respective analysis.

### Comparison of the HLA-Ligand-Atlas database with IEDB and SysteMHC

All peptides contained in the HLA Ligand Atlas database were compared with peptides listed in the IEDB and SysteMHC databases for HLA-I and HLA-II ligands separately. The list of peptides stored in the IEDB was obtained by downloading the file ‘epitope_full_v3.zip’ from the ‘Database Export’ page. The obtained table was subsequently filtered for positive MS assays, linear peptides and human origin. Peptides with modifications were removed. Peptides stored in the SysteMHC database were obtained by downloading the file ‘180409_master_final.tgz’ from ‘Builds_for_download’ page. The obtained table was subsequently filtered for human as organism.

### GO-term enrichment

GO term enrichment analyses were performed using the Panther^63^ ‘statistical overrepresentation test’ (Release 2020-07-28) based on the 2020-10-09 GO Ontology database release (DOI: 10.5281/zenodo.4081749). Gene identifiers of source proteins presented exclusively by either HLA-I or HLA-II allotypes were queried against the ‘GO cellular component complete’ database using the default ‘Homo sapiens genes’ reference list. GO terms were sorted by Fisher’s exact raw p value, and top 10 scoring terms reported as overrepresented and their corresponding p values were selected for illustration.

Tissue-specific source proteins were defined as HLA-I or HLA-II source proteins identified exclusively in one tissue across all subjects (online supplemental table S5). Gene identifiers of tissue-specific HLA-I and HLA-II source proteins were queried against the ‘GO biological process complete’ database, with the only difference that only the top five scoring terms reported as overrepresented were selected for illustration.

### Tissue-specific gene set enrichment

Analogously to the GO-term enrichment, tissue-specific HLA-I and HLA-II source proteins were separately queried against the GTEx database for gene set enrichment analysis. Gene sets with upregulated gene expression profiles per tissue ‘GTEx_Tissue_Sample_Gene_Expression_Profiles_up’ were retrieved using the gseapy implementation (V.0.9.15, 2019-08-07) through the enrichr API. All tissues covered in the HLA Ligand Atlas were matched and compared against all tissues in the GTEx database that co-occur in the HLA Ligand Atlas. Fisher’s exact raw p values for the enrichment were computed for each pairwise comparison.

### HLA-I and HLA-II peptide yield correlation to expression of immune-related genes

We computed a linear model to compare the median HLA-I peptide yields per tissue with gene expression values (RPKM) of the following genes involved in the HLA-I presentation pathway: HLA-A, HLA-B, HLA-C, immunoproteasome, constitutive proteasome, TAP1 and TAP2. Median HLA-II peptide yields per tissue were correlated to genes involved in the HLA-II presentation pathway: HLA-DRB1, HLA-DRA, HLA-DQB1, HLA-DQA1, HLA-DPB1, HLA-DPA1. The corresponding gene expression values were taken from a previously published study.^32^

An ordinary least squares linear model correlating gene expression and ${\text{log}}_{10}$ median HLA-I and HLA-II peptide yields was computed using R (V.3.5) and the corresponding stats (V.3.5) package reporting R^2^, F-statistic p value and spearman rho. The cross-correlation between all immune-related genes and their individual linear models (figure 3 and online supplemental figure S6) was computed using R (V.3.5) and the corresponding packages corrplot (V.0.84) and ggplot2 (V.3.2.1). As the expression levels of the investigated genes are highly covariant (online supplemental figure S6A, C), the regression would be overfitting when correlating peptide yields to multiple genes involved in the antigen presentation pathway, thus the analysis was limited to a single gene at a time.

### Computation of Jaccard coefficients between samples

We investigated the similarity of immunopeptidomes between tissues and subjects by pairwise comparison of all samples in the HLA Ligand Atlas. Comparisons were performed both on HLA-I and HLA-II level as well as on peptide and source protein level. The Jaccard index was calculated by dividing the set intersection by the set union for all pairwise comparisons:

$$j=\frac{A\cap B}{A\cup B}$$

### Identification of cryptic peptides with Peptide-PRISM

Identification of cryptic HLA-I peptides from HLA-I LC-MS/MS data was performed as recently described in detail.^37^ Briefly, *de novo* peptide sequencing was performed with PEAKS Studio X^64 65^ (Bioinformatics Solutions, Canada). Top 10 sequence candidates were exported for each fragment ion spectrum. Database matching of all sequence candidates and stratified FDR-filtering was performed with Peptide-PRISM using the six-frame translation of the HG38 and the three-frame translation of the human transcriptome (Ensembl 90). Matched peptides were filtered to 10% FDR and peptides were predicted as binder to the corresponding HLA alleles by NetMHCpan-4.0.^59^ Peptide-PRISM is a hybrid approach for the identification of HLA-I peptides that combines *de novo* peptide sequencing with highly efficient string search. Peptide-PRISM facilitates fast and sensitive identification of HLA-I peptides in extremely large databases that cannot be searched with classical search engines. Basically, Peptide-PRISM matches the peptide sequences of the top 10 *de novo* candidates of all fragment ion spectra against the three-frame translated human transcriptome and the six-frame translated human genome. Stringent FDR filtering is achieved by applying the common target-decoy approach in combination with mixture modeling for deconvoluting the overall *de novo* score distribution into components of false and true identifications. Since correct FDR filtering requires different *de novo* score thresholds for peptides of different length and for peptides from different categories (CDS, 5’-UTR, 3’-UTR, out-of-frame, ncRNA, intronic, intergenic), FDR filtering is performed separately for these categories (for more details see^37^). Thus, Peptide-PRISM facilitates the reliable and sensitive identification of cryptic HLA-I peptides. Due to the overall low relative abundance of cryptic peptides (~1%) in benign tissue it was not possible to reveal data about the tissue prevalence of cryptic HLA-I peptides.

### Retention time model for cryptic peptide validation

Retention time predictions were carried out using the OpenMS (V.2.5.0) RTModel based on oligo-kernel ν-support vector regression (ν=0.5, p=0.1, c=1, degree=1, border_length=22, kmer_length=1, Σ=5).^66^ The model was trained on all peptide identifications of canonical peptides identified with MHCquant and applied to all cryptic peptide identifications resulting from Peptide-PRISM. Predictions were evaluated by applying a linear least square fit to compute the 99% prediction interval around the predicted versus measured retention times using the statsmodels (V.0.11) function wls_prediction_std.

### Synthesis of isotope-labeled peptides

Peptides were synthesized using the Liberty Blue Automated Peptide Synthesizer (CEM) following the standard 9-fluorenylmethyl-oxycarbonyl/tert-butyl strategy. After removal from the resin by treatment with TFA/triisopropylsilane/water (95/2.5/2.5 by vol.) for 1 hour, peptides were precipitated from diethyl ether, washed three times with diethyl ether and resuspended in water prior to lyophilization. Purity and identity of the synthesis products were determined by C18-HPLC (Thermo Fisher Scientific, Darmstadt, Germany) and LTQ Orbitrap XL mass spectrometer (Thermo Fisher Scientific), respectively. These peptides were synthesized in house with an isotopic label, in order to avoid cross-contamination of native peptide eluates isolated in house as well with the synthesized cryptic peptides of interest.

### Spectrum validation

We selected 36 cryptic peptides, identified with an FDR of 1% for spectral validation with isotope-labeled synthetic peptides. Selected peptides were strong binders to the corresponding HLA allotype of the respective subject, with a netMHCpan-4.0 binding rank <0.5.

Isotope-labeled synthetic peptides were spiked into a sample matrix of native HLA eluted peptides from a JY cell line at a concentration of 20 fmol/µL, with the purpose of showing spectrum identity between the native and synthetic peptides.

The spectral similarity λ was computed analogous to the normalized spectral contrast angle^67^ between eluted peptide spectra and synthetic isotope labeled peptide spectra:

$$\lambda \left(S_{1},S_{2}\right)=1-\frac{2\cos^{-1}\left(S_{1}\cdot S_{2}\right)}{\pi }$$

where the spectra were encoded as intensity vectors ($S_{1}$ and $S_{2})$ based on their theoretical *b* and *y* fragment ions by using the mzR (V.2.16.2), msdata (V.0.20.0) and protViz (V.0.4) R packages. Intensities of matching y- and b-ion pairs as encoded in the intensity vectors were compared, thereby avoiding the necessity to correct for the mass shift caused by the isotope label. Peaks present in at least one of the spectra were considered for the cross product ($S_{1}∙S_{2}$). Intensities of peaks missing in one spectrum when compared with another were set to zero.

A set of 1000 randomly selected pairwise comparisons was employed to create a reference negative distribution of the spectral similarity score.

### Data storage and web interface

Data were stored and managed using the biomedical data-management platform qPortal.^68^ HLA-I and HLA-II peptides were complemented with their tissue and HLA allotype association and stored in an SQL database. A public web server was implemented that allows users to formulate queries against the database, visualize results and allows data export for further analysis. The web front-end was implemented in HTML, CSS and JavaScript based on the front-end framework Bootstrap V.4. The table plugin DataTables was used to provide rapid browsing and filtering for tabular data. Interactive plots were designed using Bokeh and ApexCharts.

## Data Availability

Data are available in a public, open access repository. All data relevant to the study are included in the article or uploaded as online supplemental information. All processed data are available in the webservice at: https://hla-ligand-atlas.org/welcome. The LC-MS/MS immunopeptidomics data comprised in the HLA Ligand Atlas has been deposited to the ProteomeXchange Consortium via the PRIDE (Perez-Riverol *et al*) partner repository with the dataset identifier PXD019643 and the project DOI 10.6019/PXD019643. LC-MS/MS runs and samples not adhering to the implemented quality control thresholds are deposited as well. The LC-MS/MS immunopeptiodmics data from the three glioblastoma patients can be accessed with the identifier PXD020186, and the project DOI 10.6019/PXD020186.
