# Supplementary material for: HLA Ligand Atlas: a benign reference of HLA-presented peptides to improve T-cell-based cancer immunotherapy
Source: J Immunother Cancer. 2021 Apr 15;9(4):e002071. doi: 10.1136/jitc-2020-002071 (PMC8054196; doi:10.1136/jitc-2020-002071)
Supplement: Supplementary data [file jitc-2020-002071supp001.pdf]

BMJ Publishing Group Limited (BMJ) disclaims all liability and responsibility arising from any reliance placed on this supplemental material which has been supplied by the author(s)

The HLA Ligand Atlas: A benign reference of HLA-presented peptides to improve T-cell-based cancer immunotherapy

Ana Marcu[§], Leon Bichmann[§], Leon Kuchenbecker[§], Daniel Johannes Kowalewski, Lena Katharina Freudenmann, Linus Backert, Lena Mühlenbruch, András Szolek, Maren Lübke, Philipp Wagner, Tobias Engler, Sabine Matovina, Jian Wang, Mathias Hauri-Hohl, Roland Martin, Konstantina Kapolou, Juliane Sarah Walz, Julia Velz, Holger Moch, Luca Regli, Manuela Silginer, Michael Weller, Markus W. Löffler, Florian Erhard, Andreas Schlosser, Oliver Kohlbacher, Stefan Stevanović, Hans-Georg Rammensee, Marian Christoph Neidert

[§]these authors contributed equally to this work

Supplementary FIGURES

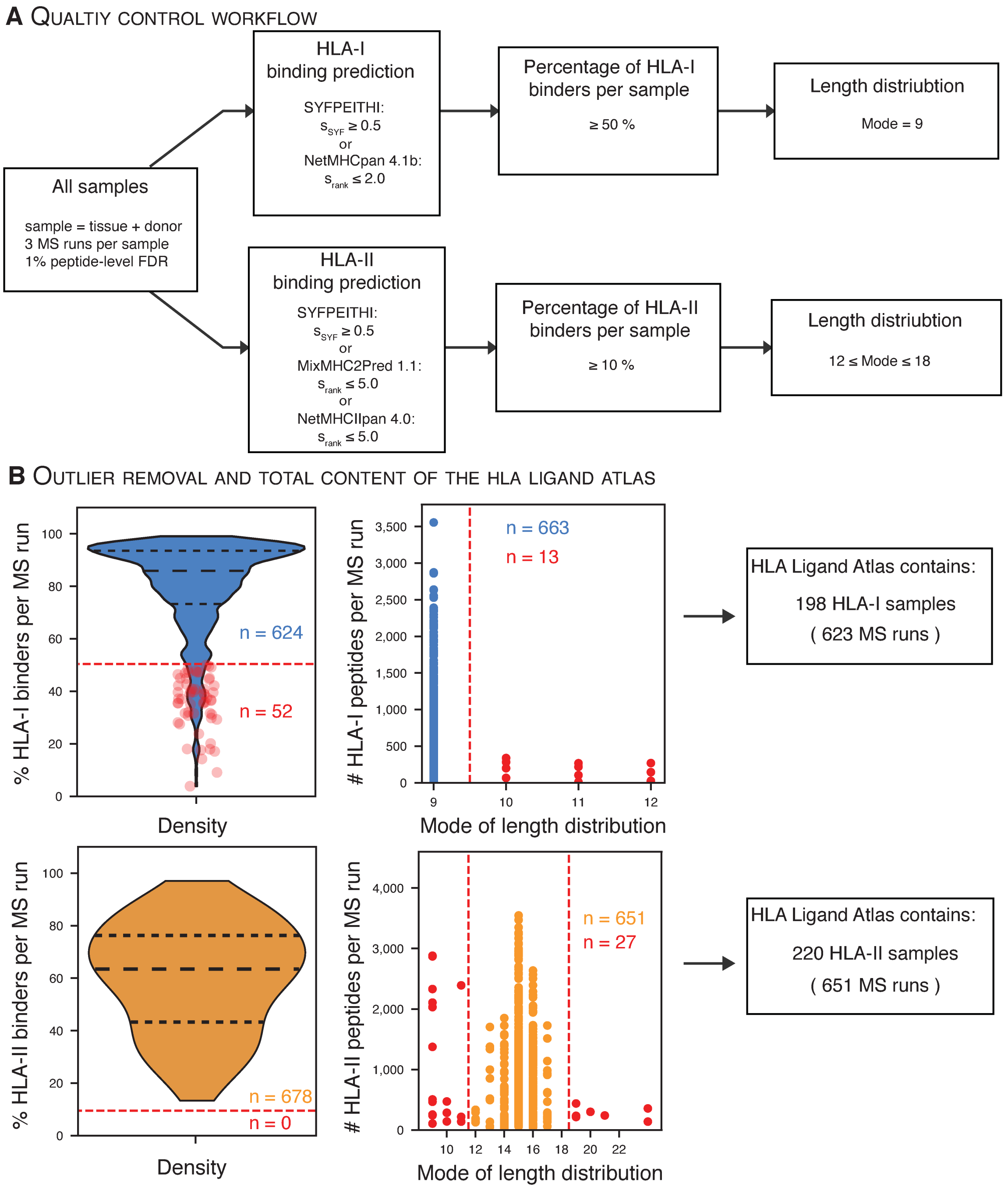
A QUALTIY CONTROL WORKFLOW
All samples
sample = tissue + donor
3 MS runs per sample
1% peptide-level FDR
HLA-I binding prediction
SYFPEITHI: s_SYF ≥ 0.5
or
NetMHCpan 4.1b: s_rank ≤ 2.0
Percentage of HLA-I binders per sample
≥ 50 %
Length distriubtion
Mode = 9
HLA-II binding prediction
SYFPEITHI: s_SYF ≥ 0.5
or
MixMHC2Pred 1.1: s_rank ≤ 5.0
or
NetMHCIIpan 4.0: s_rank ≤ 5.0
Percentage of HLA-II binders per sample
≥ 10 %
Length distriubtion
12 ≤ Mode ≤ 18
B OUTLIER REMOVAL AND TOTAL CONTENT OF THE HLA LIGAND ATLAS
% HLA-I binders per MS run
n = 624
n = 52
Density
# HLA-I peptides per MS run
n = 663
n = 13
Mode of length distribution
HLA Ligand Atlas contains:
198 HLA-I samples
(623 MS runs)
% HLA-II binders per MS run
n = 678
n = 0
Density
# HLA-II peptides per MS run
n = 651
n = 27
Mode of length distribution
HLA Ligand Atlas contains:
220 HLA-II samples
(651 MS runs)

Supplementary Figure S1: Computational workflow and quality control thresholds (related to Figure 1)
(A) Data from three LC-MS/MS runs (technical replicates) per sample (one tissue from one subject) were processed with MHCquant with a local peptide-level FDR of 1%. Identified peptides were categorized into peptides predicted as strong, weak, or non-binders. HLA-I peptides were predicted by SYFPEITHI and NetMHCpan 4.0, employing a cut-off threshold of $s_{\text{SYF}} \geq 0.5$ and $s_{\text{rank}} \leq 2.0$, respectively. HLA-II peptides were predicted with MixMHCPred, NetMHCIIpan 4.0 and SYFPEITHI with equal thresholds. Samples with less than 50% and 10% predicted binders per technical replicate for HLA-I and HLA-II immunopeptidomes were excluded from further analysis and are not included in the HLA Ligand Atlas database release. Finally, we employed a stringent cut-off concerning the mode of the peptide length distribution for HLA-I (mode equal to 9) and -II (mode between 12 to18) immunopeptidomes.
(B) LC-MS/MS runs not pertaining to the aforementioned QC thresholds (dashed red lines) concerning the percentage of predicted HLA-binders and the peptide length distribution were not included in the HLA Ligand Atlas database release: Violin plots (left) depict the percentage of peptides predicted to be HLA-binders per LC-MS/MS run and the quality control cut-off for LC-MS/MS runs employed for HLA-I and -II immunopeptidomes. Dot plots (center) depict the mode of the peptide length distribution encountered per LC-MS/MS run and the quality control cut-off employed for HLA-I and -II. The number of LC-MS/MS runs (HLA-I - blue and HLA-II orange) failing the QC thresholds is indicated by red dots. The final number of LC-MS/MS runs and samples selected for inclusion into the HLA Ligand Atlas database release and data analysis are indicated on the right and differ for HLA-I and –II experiments.

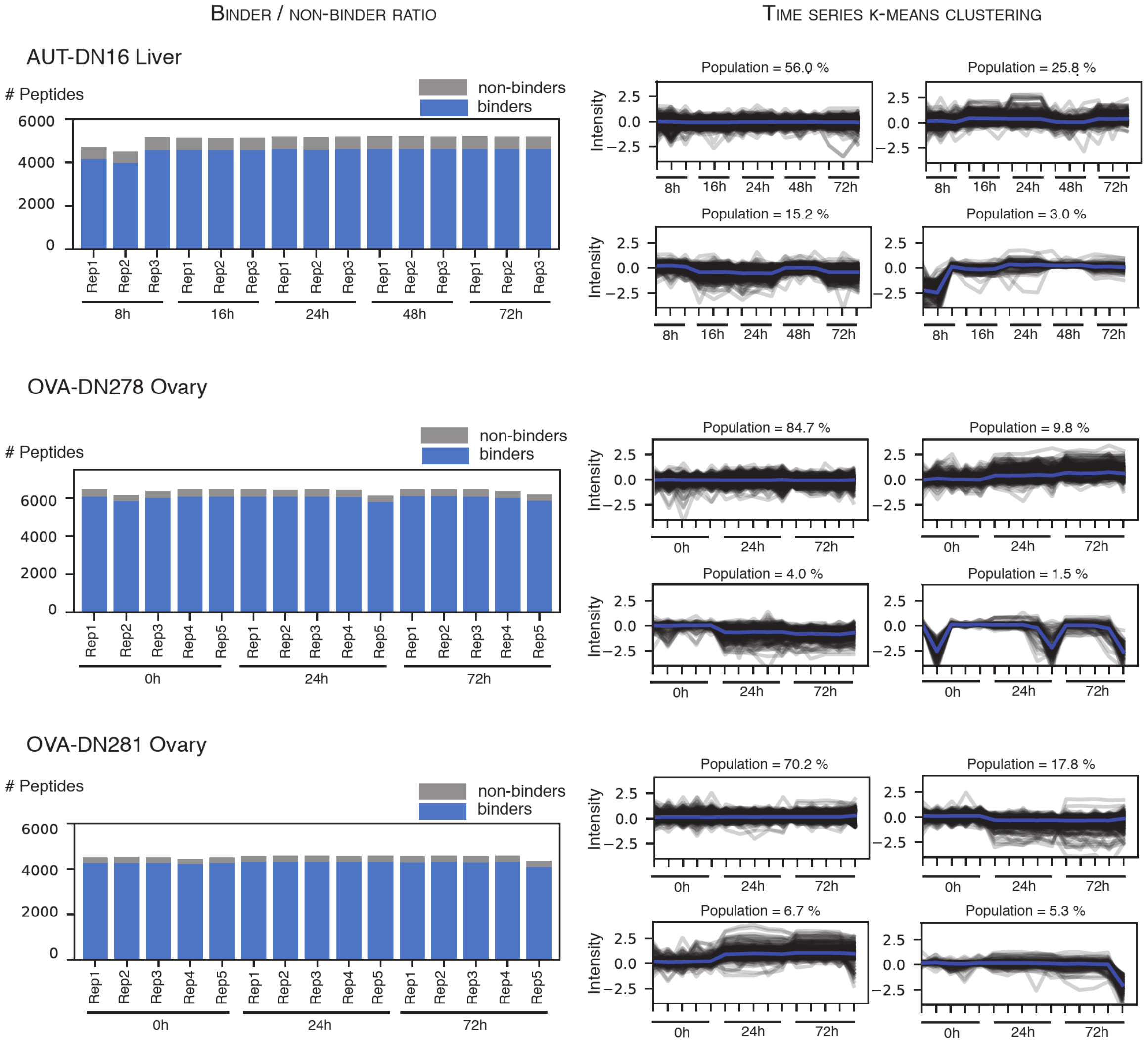

Supplementary Figure S2: Time-Series Experiment (related to Figure 1)
The time series experiment was carried out on three biological samples from three subjects. Bar plots (left hand side) indicate the number of identified HLA-I predicted binders (blue) and predicted non-binders (grey) across technical replicates and timepoints for each timeseries. Time series (right hand side) indicate individual clusters (K-Means using four seeds) of trajectories of quantified MS1 intensity across technical replicates and timepoints. Intermediate trend lines for each cluster are indicated in blue and the percentage of trajectories populating a given cluster is annotated at the top edge of each plot. The trajectories of all timepoints were set in relation to the initial timepoint. The analysis reveals that the number of identified peptides and their percentage of predicted HLA-binders is constant and that most trajectories do not vary much across time points.

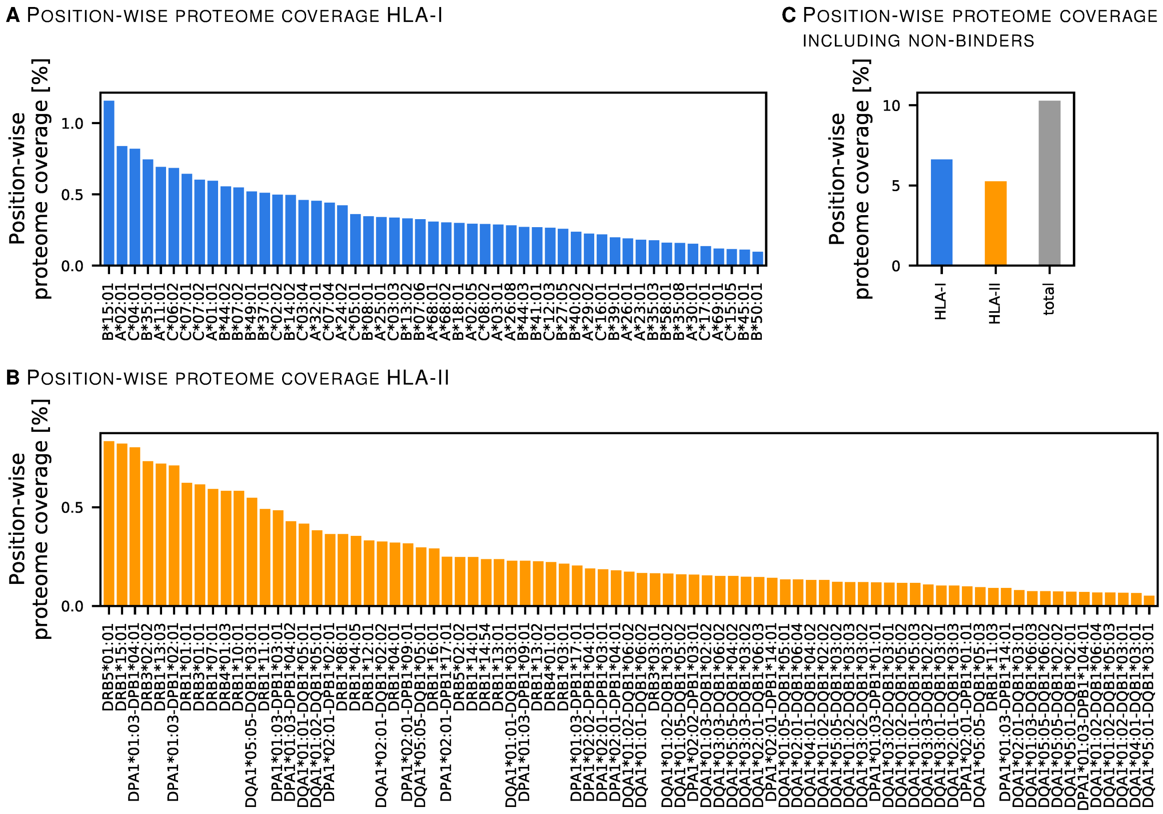

Supplementary Figure S3: Position-wise proteome coverage (related to Figure 1C, Figure 2B and C)
(A-B) Analysis of the different propensities of HLA-I (blue) and –II (orange), allotypes concerning the presentation of peptides. In turn different percentages of the source proteome are position-wise covered. For example, HLA-B*15:01 ligands cover over 1% of the HLA-I source proteome (see Figure 2B, C).
(C) Global analysis of the position-wise coverage of all proteins presented on either HLA-I (blue), HLA-II (orange), or both independent of specific allotypes (see Figure 1C).

Marcu A, *et al. J Immunother Cancer* 2021; 9:e002071. doi: 10.1136/jitc-2020-002071

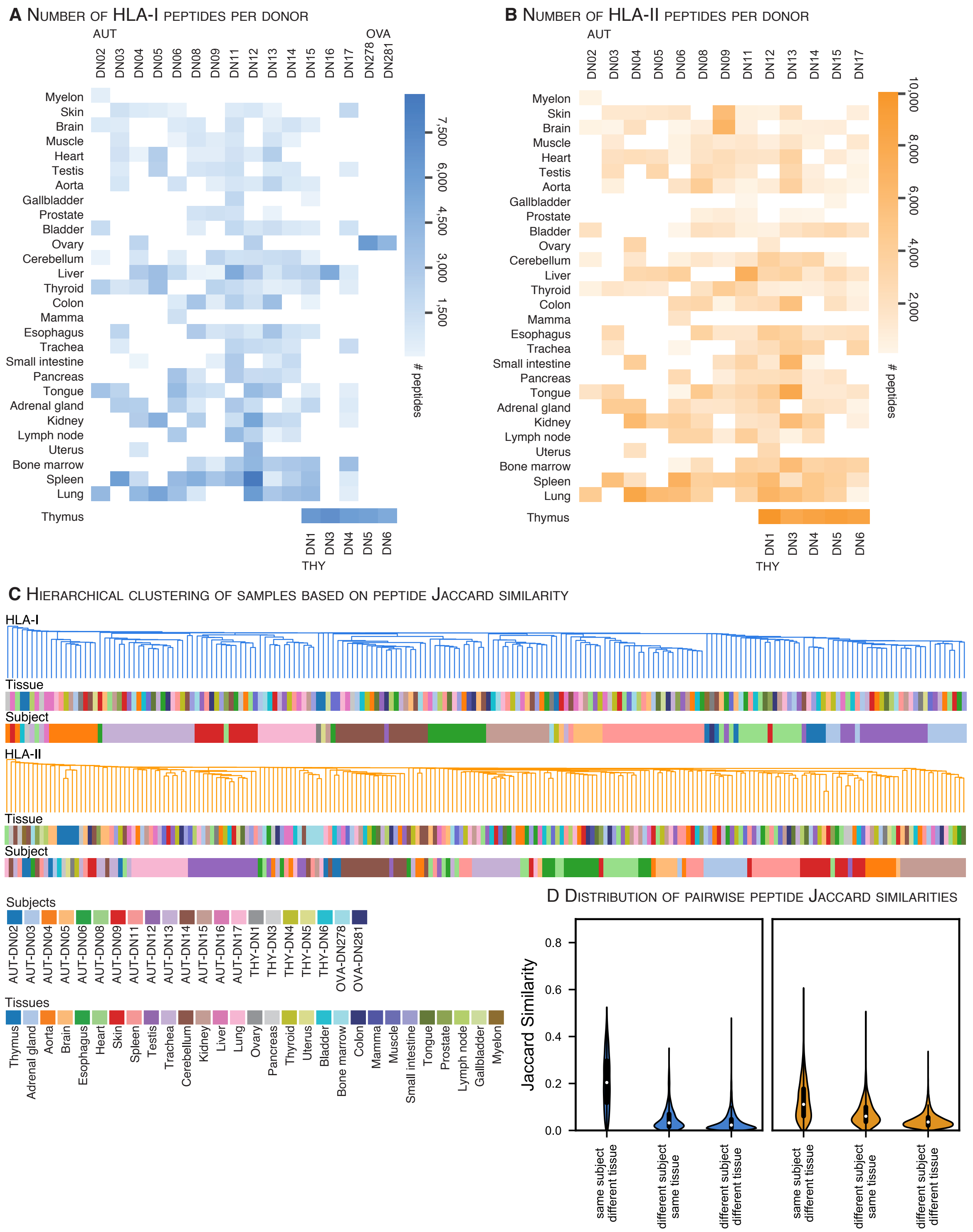

Marcu A, et al. J Immunother Cancer 2021; 9:e002071. doi: 10.1136/jitc-2020-002071

BMJ Publishing Group Limited (BMJ) disclaims all liability and responsibility arising from any reliance placed on this supplemental material which has been supplied by the author(s)

Supplementary Figure S4: Distribution of peptide identifications and yields across tissues and subjects and peptide-based hierarchical sample clustering (related to Figure 2D, E, and Figure 4A)

(A-B) HLA-I peptide yields per tissue and subject are illustrated in a heatmap for HLA-I and –II. The color range is in accordance with the number of peptides identified in each sample as indicated in the legend on the right (HLA-I – blue, HLA-II – orange).

(C) Pairwise hierarchical clustering of samples based on the Jaccard similarity between HLA-I (blue) and HLA-II (orange) peptides. The dendrogram illustrates the nearest neighbor based on the similarity between tissues and subjects. See Figure 2D.

(D) Violin plots illustrate the distribution of the peptide Jaccard similarity index for each pairwise comparison between the same subject - different tissues; different subjects - the same tissue, and different subject - different tissues. Results confirmed for HLA-I and -II source proteins Figure 2D and E, respectively.

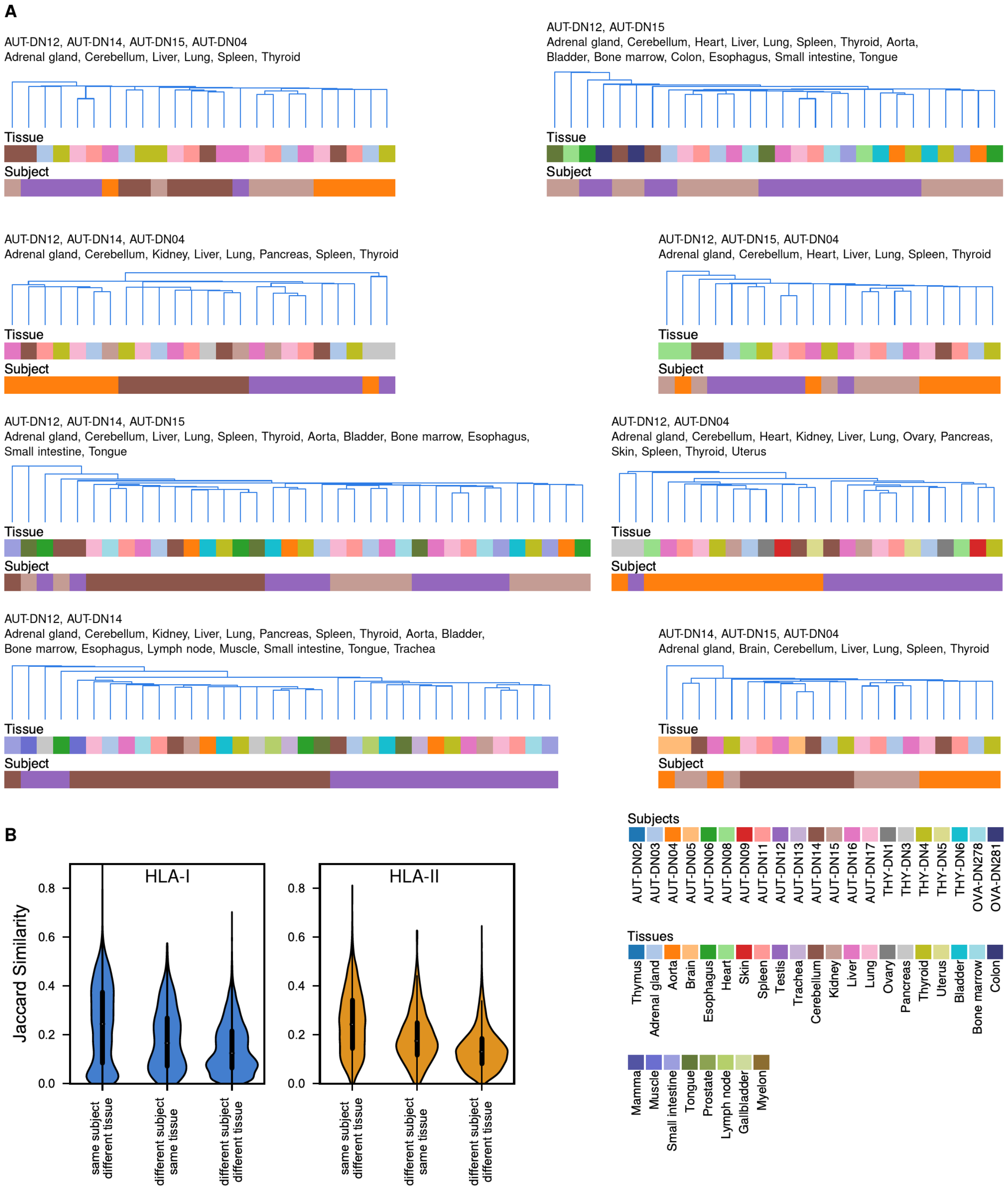
A
AUT-DN12, AUT-DN14, AUT-DN15, AUT-DN04
Adrenal gland, Cerebellum, Liver, Lung, Spleen, Thyroid
AUT-DN12, AUT-DN15
Adrenal gland, Cerebellum, Heart, Liver, Lung, Spleen, Thyroid, Aorta, Bladder, Bone marrow, Colon, Esophagus, Small intestine, Tongue
AUT-DN12, AUT-DN14, AUT-DN04
Adrenal gland, Cerebellum, Kidney, Liver, Lung, Pancreas, Spleen, Thyroid
AUT-DN12, AUT-DN15, AUT-DN04
Adrenal gland, Cerebellum, Heart, Liver, Lung, Spleen, Thyroid
AUT-DN12, AUT-DN14, AUT-DN15
Adrenal gland, Cerebellum, Liver, Lung, Spleen, Thyroid, Aorta, Bladder, Bone marrow, Esophagus, Small intestine, Tongue
AUT-DN12, AUT-DN04
Adrenal gland, Cerebellum, Heart, Kidney, Liver, Lung, Ovary, Pancreas, Skin, Spleen, Thyroid, Uterus
AUT-DN12, AUT-DN14
Adrenal gland, Cerebellum, Kidney, Liver, Lung, Pancreas, Spleen, Thyroid, Aorta, Bladder, Bone marrow, Esophagus, Lymph node, Muscle, Small intestine, Tongue, Trachea
AUT-DN14, AUT-DN15, AUT-DN04
Adrenal gland, Brain, Cerebellum, Liver, Lung, Spleen, Thyroid
Tissue
Subject
B
HLA-I
HLA-II
Jaccard Similarity
0.0
0.2
0.4
0.6
0.8
same subject different tissue
different subject same tissue
different subject different tissue
Subjects
AUT-DN02
AUT-DN03
AUT-DN04
AUT-DN05
AUT-DN06
AUT-DN08
AUT-DN09
AUT-DN11
AUT-DN12
AUT-DN13
AUT-DN14
AUT-DN15
AUT-DN16
AUT-DN17
THY-DN1
THY-DN3
THY-DN4
THY-DN5
THY-DN6
OVA-DN278
OVA-DN281
Tissues
Thymus
Adrenal gland
Aorta
Brain
Esophagus
Heart
Skin
Spleen
Testis
Trachea
Cerebellum
Kidney
Liver
Lung
Ovary
Pancreas
Thyroid
Uterus
Bladder
Bone marrow
Colon
Mamma
Muscle
Small intestine
Tongue
Prostate
Lymph node
Gallbladder
Myelon

Supplementary Figure S5: Hierarchical sample clustering for HLA and tissue subset matched sample groups (related to Figure 2D, E)

(A) Hierarchical clustering of predicted HLA-A*02:01 binding peptide sets based on their Jaccard similarity. Samples were selected from donors carrying the HLA-A*02:01 allele and subsequently filtered for predicted binders against that allele. The samples were then divided into subsets of donors and tissues such that ever tissue was measured in every donor. The resulting subsets of samples were then clustered as described in the Methods section.

(B) The distributions of Jaccard similarity scores for equivalently HLA allele matched sample pairs over all HLA allotypes present in the dataset.

Marcu A, *et al. J Immunother Cancer* 2021; 9:e002071. doi: 10.1136/jitc-2020-002071

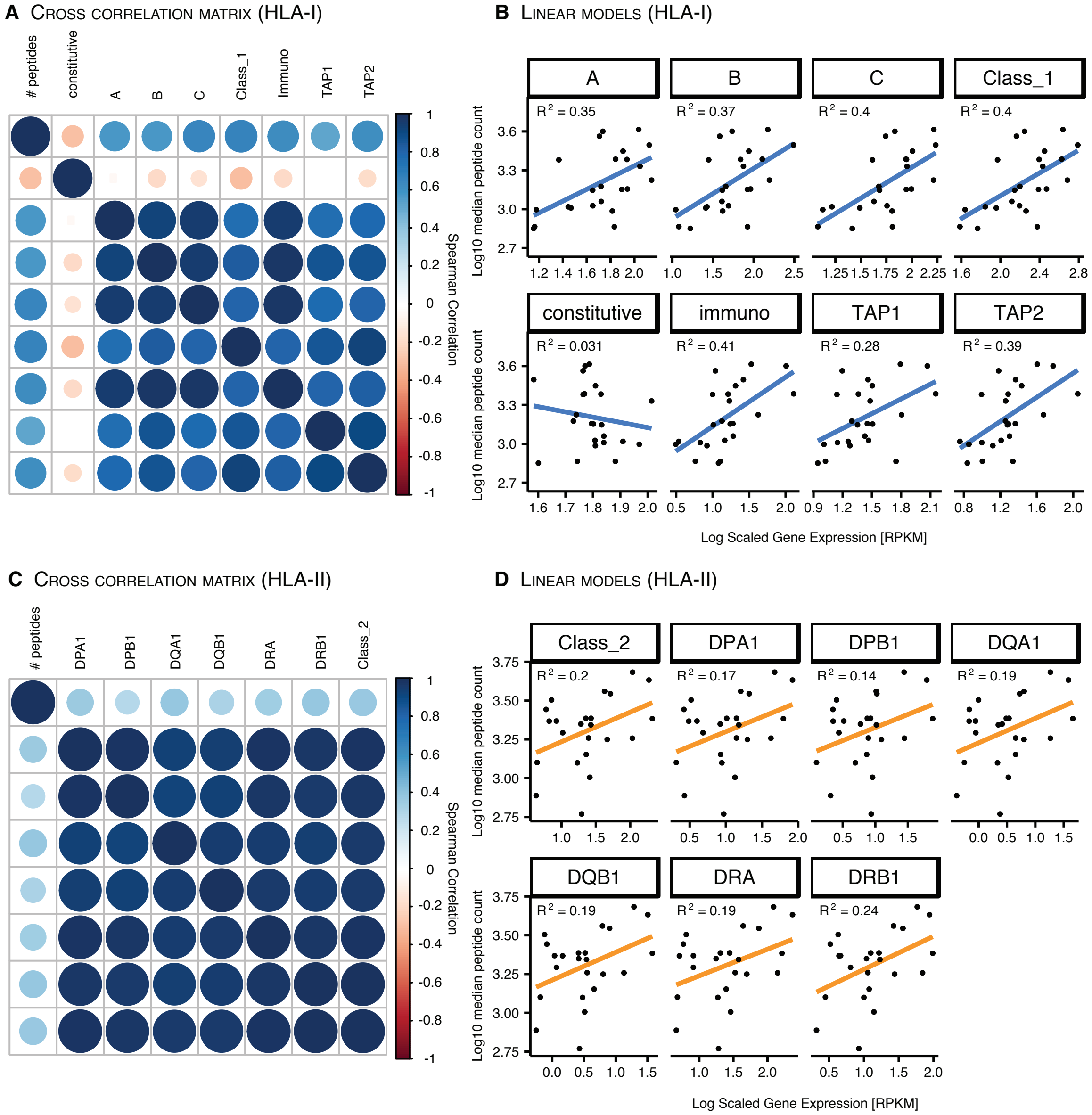
A Cross correlation matrix (HLA-I)
B Linear models (HLA-I)
C Cross correlation matrix (HLA-II)
D Linear models (HLA-II)
Spearman Correlation
Log10 median peptide count
Log Scaled Gene Expression [RPKM]

Supplementary Figure S6: Linear models of gene expression across tissues (related to Figure 3). The correlation of HLA-I and -II peptide yields across tissues was assessed with respect to the expression levels (RPKM) of multiple immune related genes of the HLA-I and -II antigen presentation pathway. (RNA expression values were taken from a different publication (Boegel et al., 2018).

(A,C) Symmetrical cross-correlation matrices were generated, illustrating the spearman correlation coefficients (rho) of the number of identified HLA-I and -II ligands with expression levels (RPKM) of relevant genes in the HLA-I and -II antigen presentation pathways and among each other. The color-coded dots and their size represent the degree (Spearman rho) of positive (blue) or negative correlation (red).

(B,D) In addition, linear models illustrate HLA-I and -II immunopeptidome yields correlated to log scaled gene expression values (RPKM) of genes involved in the HLA-I and -II antigen processing pathway, respectively. The R2 correlation coefficient is depicted in the top left corner for each comparison.

EVVTEVVSR, AUT01-DN08 Spleen

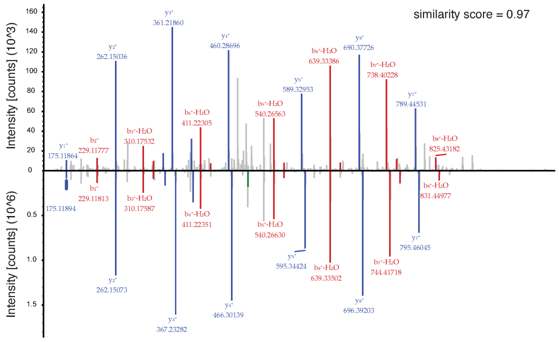

EVVTEVVSR, V7-Label13C(5)15N(1) (6.01381 Da)

IYPPLLGALSF, AUT01-DN09 Bone Marrow

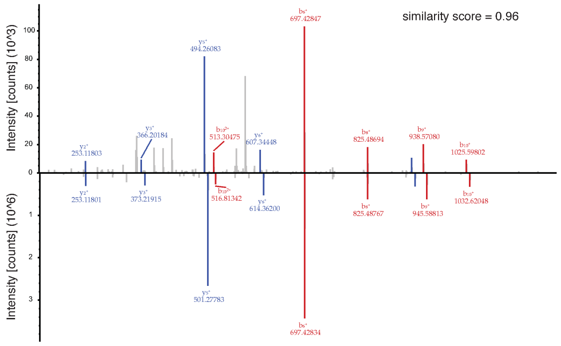

IYPPLLGALSF, L9-Label13C(6)15N(1) (7.01716 Da)

RPFSPTLRVL, AUT01-DN06 Tongue

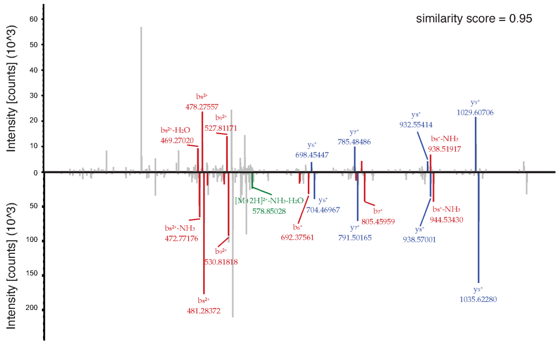

RPFSPTLRVL, P5-Label13C(5)15N(1) (6.01381 Da)

RPRPPPATTL, AUT01-DN06 Adrenal Gland

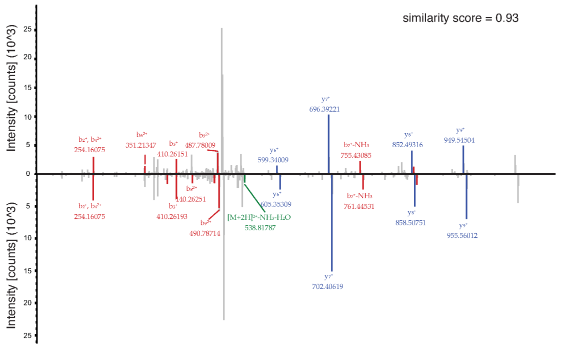

RPRPPPATTL, P6-Label13C(5)15N(1) (6.01381 Da)

HPRTIQGKL AUT01-DN06 Lung

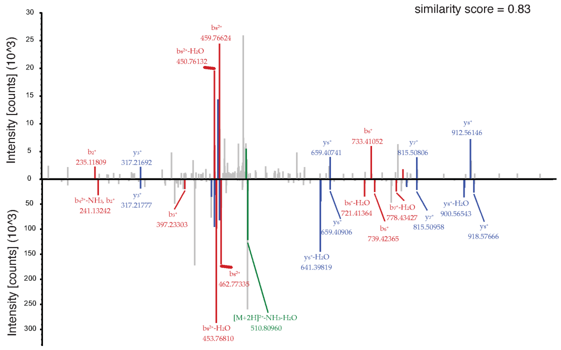

HPRTIQGKL, P2-Label13C(5)15N(1) (6.01381 Da)

STIHLASQFTK, AUT01-DN08 Colon

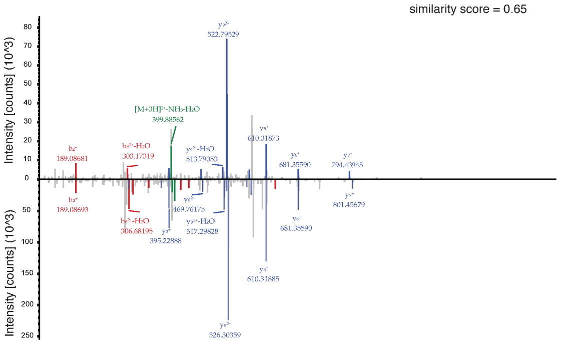

STIHLASQFTK, L5-Label13C(6)15N(1) (7.01716 Da)

Marcu A, *et al. J Immunother Cancer* 2021; 9:e002071. doi: 10.1136/jitc-2020-002071

Supplementary Figure S7: Manual validation of cryptic peptide identifications with isotope labeled synthetic peptides (related to Figure 6). Six exemplary spectral comparisons depict cryptic peptides identified in diverse tissues and subjects (upper spectrum) related to their synthetic isotope labeled counterpart (lower spectrum). The isotope labeled amino acid is highlighted in red and its corresponding label and mass are annotated. Matching b- (red), y-ions (blue) and neutral losses (green) and their corresponding fragment masses are annotated to each peak in the spectra. The spectral similarity score of the given comparison is annotated in the top right.
